# MTR4 drives liver tumorigenesis by promoting cancer metabolic switch through alternative splicing

**DOI:** 10.1038/s41467-020-14437-3

**Published:** 2020-02-05

**Authors:** Lili Yu, Jinchul Kim, Lei Jiang, Bingbing Feng, Yue Ying, Kai-yuan Ji, Qingshuang Tang, Wancheng Chen, Taoyi Mai, Wenlong Dou, Jianlong Zhou, Le-yang Xiang, Yang-fan He, Dinghua Yang, Qingjiao Li, Xuemei Fu, Yang Xu

**Affiliations:** 1 The Eighth Affiliated Hospital, Sun Yat-sen University, Shenzhen, Guangdong 518033 China; 2 Cancer Research Institute, Guangdong Provincial Key Laboratory of Cancer Immunotherapy, School of Basic Medical Sciences, Southern Medical University, Guangzhou, Guangdong 510515 China; 3 Division of Biological Sciences, University of California, San Diego, 9500 Gilman Drive, La Jolla, CA 92093-0322 USA; 4 Department of Hepatobiliary Surgery, Nanfang Hospital, Southern Medical University, Guangzhou, Guangdong 510515 China; 5 Department of Pathology, State Key Laboratory of Oncology in South China, Collaborative Innovation Center for Cancer Medicine, Sun Yat-sen University Cancer Center, Guangzhou, Guangdong 510060 China; 6 Shenzhen Children’s Hospital, 7019 Yitian Road, Shenzhen, 518026 China

**Keywords:** Cancer metabolism, Gastrointestinal cancer

## Abstract

The metabolic switch from oxidative phosphorylation to glycolysis is required for tumorigenesis in order to provide cancer cells with energy and substrates of biosynthesis. Therefore, it is important to elucidate mechanisms controlling the cancer metabolic switch. MTR4 is a RNA helicase associated with a nuclear exosome that plays key roles in RNA processing and surveillance. We demonstrate that MTR4 is frequently overexpressed in hepatocellular carcinoma (HCC) and is an independent diagnostic marker predicting the poor prognosis of HCC patients. MTR4 drives cancer metabolism by ensuring correct alternative splicing of pre-mRNAs of critical glycolytic genes such as *GLUT1* and *PKM2*. c-Myc binds to the promoter of the MTR4 gene and is important for MTR4 expression in HCC cells, indicating that MTR4 is a mediator of the functions of c-Myc in cancer metabolism. These findings reveal important roles of MTR4 in the cancer metabolic switch and present MTR4 as a promising therapeutic target for treating HCC.

## Abstract (web summary)

Aberrant alternative splicing has been shown to contribute to the tumorigenic processes. Here, the authors show that MTR4 is overexpressed in hepatocellular carcinoma and has a role in tumorigenesis through the modulation of the splicing of glycolytic genes *PKM2* and *GLUT1*.

## Introduction

Hepatocellular carcinoma (HCC) has become one of the major cancers in the world with limited therapeutic options and increasing mortality rate^1^. In contrast to normal somatic cells that obtain energy primarily from oxidative phosphorylation (OXPHOS), human cancer cells depend on glycolysis to obtain energy and substrates for biosynthesis^2^. Therefore, the elucidation of the pathways important for cancer metabolic switch from OXPHOS to glycolysis will provide more specific therapeutic targets for cancer therapy. While many oncogenic pathways such as c-Myc pathway play important roles in promoting cancer metabolic switch by regulating the expression of metabolic genes^3^, various types of human cancers exhibit high levels of heterogeneity and may develop distinct mechanisms to induce cancer metabolic reprogramming. For example, recent studies demonstrate that HCC cells expressing wild type *p53* depend on p53-PUMA pathway to maintain the metabolic switch from OXPHOS to glycolysis through PUMA-mediated disruption of mitochondrial pyruvate carrier-dependent pyruvate uptake^4^.

The regulation of gene expression can be achieved at many levels, including the transcriptional and posttranscriptional mechanisms. The nuclear exosome monitors and degrades RNAs using RNA-binding cofactor complexes that recruit specific RNA targets for processing. Two major cofactor complexes, the cytoplasmic Ski and nuclear TRAMP complexes, are involved in recruiting RNAs to exosome^5^. RNA helicases such as MTR4 are associated with cofactor complexes and unwind complex RNA structures to permit the insertion of RNA into exosome for processing^5^. MTR4 is present in the TRMAP complex, the nuclear exosome targeting (NEXT) complex and poly-A tail exosome targeting (PAXT) complex for RNA decay, suggesting that MTR4 targets a broad spectrum of RNA and regulates their stability. MTR4 has not been shown to play important roles in alternative splicing (AS), but the exosome is involved in chromatin remodeling and normal processing of various RNAs such as pre-mRNA splicing^6–9^. While the biochemical functions of MTR4 have been extensively investigated, the physiological roles of MTR4 in development and disease remain unclear.

Recent studies have demonstrated that various types of human cancer could develop aberrant AS landscape, contributing to the tumorigenic processes^10^. These cancer relevant AS patterns in human cancer cells could result from the mutations in splicing sites of pre-mRNAs and regulatory elements or alterations in spliceosome components^11^. For example, the abnormal regulation of the AS events of the key metabolic genes such as *PKM1*/*PKM2* could drive cancer metabolic reprogramming^12^. Therefore, to improve the specificity and efficacy of current therapeutic interventions, it is important to elucidate the mechanisms underlying the aberrant RNA splicing in order to identify new targets for suppressing cancer relevant splicing events. Polypyrimidine tract-binding protein 1 (PTBP1) is an important regulator of AS^13^. The binding of PTBP1 to pre-mRNA suppresses the splicing of exons adjacent to the binding site. However, the sequence of the binding sites of PTBP1 to pre-mRNA is conserved and cannot explain the aberrant splicing events in cancer cells. Therefore, it is important to identify PTBP1-interacting factors in cancer cells that could modulate the interaction between the pre-mRNA and PTBP1, leading to aberrant cancer-specific splicing events.

**Fig. 1:**
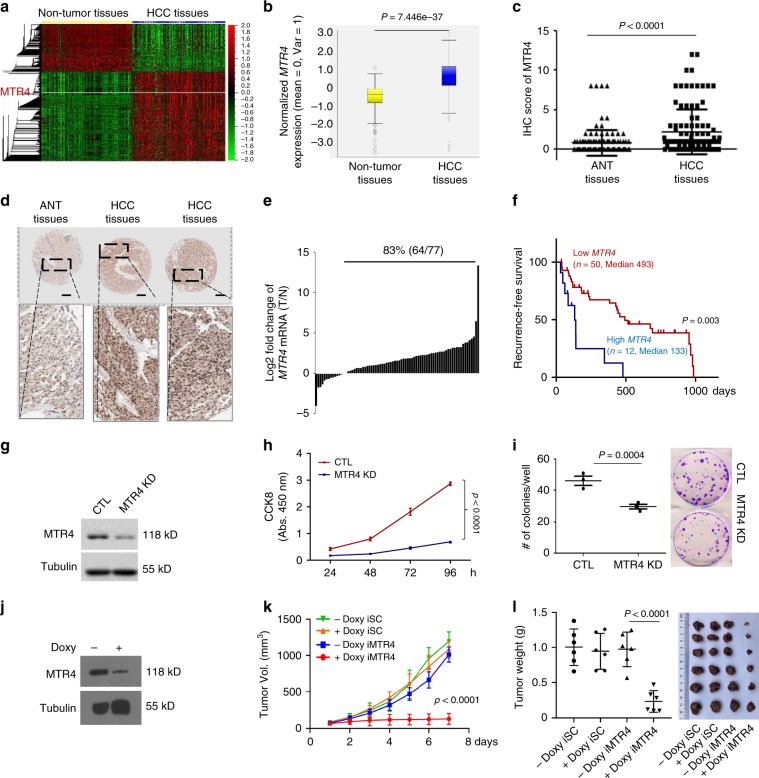
MTR4 is required for the tumorigenesis of HCC cells. **a** Heat map of the global mRNA expression profile in non-tumor tissues (*n* = 220) and hepatocellular carcinoma (HCC) tissues (*n* = 225) in GSE14520 dataset. The levels of *MTR4* are indicated with a white line. **b** Box plot showing the relative mRNA levels of *MTR4* in HCC tissues (*n* = 225) and non-tumor tissues (*n* = 220) in GSE14520 (GPL3921) dataset. The significance of the difference was assessed by two-tailed, unpaired *t*-test. *p* value is indicated. Centre is median within box, bound of the box spans the interquartile range, and whiskers visualize 5 and 95% of the data points. **c**, **d** Chips of HCC samples (*n* = 108) and adjacent non-tumor (ANT) samples (*n* = 108) were stained with anti-MTR4 antibody and the intensity of staining was scanned and scored. Representative immunohistochemistry (IHC) images are shown. Two-tailed, unpaired *t*-test. Data are presented as mean value ± s.d. *p* value is indicated. Scale bar = 200 µm. **e** The *MTR4* mRNA levels in HCC samples (*n* = 77) were compared with the corresponding ANT samples (*n* = 77). Eighty-three percent (64/77) of HCC samples have higher *MTR4* mRNA levels than ANT. **f** The *MTR4* levels were inversely correlated with the postoperative recurrence-free survival (RFS) of HCC patients. RFS of the patients with high *MTR4* mRNA levels (*n* = 12) is significantly lower than those with lower *MTR4* (*n* = 50). The difference in survival rates was assessed with the log-rank test (Mantel Cox). *p* value is indicated. **g** The knockdown of MTR4 in HCC cells PLC/PRF/5 was confirmed by western blotting. CTL, HCC cells expressing scramble shRNA. Tubulin was used as an internal control. Consistent data were obtained from two independent experiments. **h** Proliferation of MTR4 KD and control cells was analyzed with CCK8 assay. *n* = 3 biologically independent experiments. Difference between two groups was calculated by two-way ANOVA, followed by Bonferroni post-tests. Data are presented as mean value ± s.d. *p* value is indicated. **i** Colony formation assay of the control and MTR4 KD cells. Difference between two groups was calculated by two-tailed, unpaired *t*-test. Data are presented as mean value ± s.d. *p* value is indicated. *n* = 3 biologically independent samples. **j** Inducible knockdown of MTR4 in PLC/PRF/5 cells (iMTR4 cells) was confirmed by western blotting after the treatment with 1 µg/ml doxycycline (Doxy) for 4 days. Consistent data were obtained from two independent experiments. **k**, **l** The volumes (**k**) and weight (**l**) of tumors formed by PLC/PRF/5 cells expressing scramble shRNA (iSC) or iMTR4 cells in NSG mice were measured after daily i.p. injection of Doxy (20 mg kg^−1^ body weight) or mock treatment for 8 days. Individual tumor volumes were measured every day after doxy treatment. Repeated measures two-way ANOVA, followed by Turkey’s post-tests. *p* value is indicated. Repeated measures two-way ANOVA, followed by Bonferroni post-tests. Data are presented as mean value ± s.d. *p* value is indicated. *n* = 6 independent samples for each group. At the end of the treatment, the weight of all tumors in each group was compared. Mann–Whitney test. *p* value is indicated. *n* = 6 independent samples for each group. Source data are provided as a Source Data file.

To identify the proteins that might be important for the aberrant AS and tumorigenesis of HCC, we analyzed the transcriptional dataset assembled from 225 HCC tissues and 220 non-cancerous liver tissues available in the Gene Expression Omnibus (GEO) database (http://www.ncbi.nlm.nih.gov/geo/) and discovered that the mRNA levels of the RNA helicase MTR4 were increased in HCC tissues (Fig. 1a, b). We demonstrated the important roles of MTR4 in promoting HCC tumorigenesis and cancer metabolic reprogramming by regulating HCC relevant AS events through recruiting PTBP1 to its target pre-mRNAs. Our findings reveal the mechanisms underlying aberrant AS events and cancer metabolic reprogramming in HCC, and provide a new therapeutic target for treating HCC by inhibiting HCC relevant AS.

## Results

### MTR4 is required for the tumorigenesis of HCC cells

Consistent with the mRNA expression data, immunohistochemical analysis of MTR4 in 108 paired HCC tissues and adjacent non-tumor (ANT) tissues indicated that the protein levels of MTR4 are significantly higher in HCC samples than in ANT tissues (Fig. 1c, d, Supplementary Fig. 1). To reveal the clinical relevance of the expression levels of MTR4 in HCC, we analyzed the correlation between the expression levels of MTR4 in the resected HCC tissues and the prognosis of corresponding HCC patients, indicating that the expression levels of MTR4 in HCC tissues are inversely correlated with the recurrence-free survival (RFS) of HCC patients (Fig. 1e, f). Univariate analysis indicates that the high expression level of MTR4 is significantly associated with four-pathological markers of HCC such as tumor size (*p* = 0.027), tumor number (*p* = 0.006), MVI (*p* < 0.001), and differentiation (*p* = 0.043) (Table 1). Analysis with Multivariate Cox proportional hazards model indicates that the expression levels of MTR4 is an independent prognostic marker for RFS of HCCs, after the adjustment for tumor size, tumor number, MVI, and differentiation (Table 1).

**Table 1:** Univariate and multivariate Cox regression analyses of recurrence-free survival for MTR4 (*n* = 62).

Variables	HR	95% CI	*P* value
*Univariate analysis*			
Age (<60 vs ≥60 years)	0.385	0.144–1.027	0.057
Gender (male vs female)	0.535	0.186–1.536	0.245
AFP (<400 vs ≥400)	1.691	0.853–3.352	0.133
Liver cirrhosis (yes vs no)	0.859	0.375–1.966	0.719
Capsule (yes vs no)	0.913	0.374–2.232	0.843
Tumor size (<5 vs ≥5 cm)	2.304	1.097–4.837	0.027
Tumor number (*n* = 1 vs *n* ≥ 2)	3.310	1.412–7.756	0.006
MVI^a^ (yes vs no)	4.697	2.094–10.536	<0.0001
PVTT^b^ (yes vs no)	1.982	0.948–4.144	0.069
Differentiation (well, moderate, poor)	0.527	0. 283–0.980	0.043
Expression of MTR4 (high vs low)	4.081	1.780–9.353	0.0001
*Multivariate analysis*			
Tumor number	5.130	1.992–13.207	0.001
MVI (yes vs no)	7.358	2.799–19.341	0.000
PVTT (yes vs no)	0.472	0.195–1.144	0.682
MTR4 expression (high vs low)	5.414	1.968–14.891	0.001
Size_5	2.147	0.945–4.875	0.068
Differentiation	1.471	0.720–3.006	0.289

^a^Micro-vascular invasion.

^b^Portal vein tumor thrombosis.

To investigate the roles of MTR4 in the tumorigenesis of HCC, we silenced the expression of MTR4 in two HCC cell lines, PLC/PRF/5 cells and HepG2 cells, with distinct shRNAs (Fig. 1g, Supplementary Fig. 2). The knockdown of MTR4 significantly reduces the proliferation and colony-forming ability of HCC cells (Fig. 1h, i, Supplementary Fig. 2b, c). Inducible knockdown of MTR4 in HCC tumors already established in the immunodeficient NODSCID mice greatly suppresses tumor growth (Fig. 1j–l, Supplementary Fig. 2e, f). In contrast, doxycycline (Doxy) at the given dosage has little effect on the tumor growth of HCC cells expressing the scramble shRNA inducibly in NODSCID mice (Fig. 1k, l). These data demonstrate that MTR4 is required for the tumorigenesis of HCC cells.

### MTR4 is required to maintain glycolysis of HCC cells

**Fig. 2:**
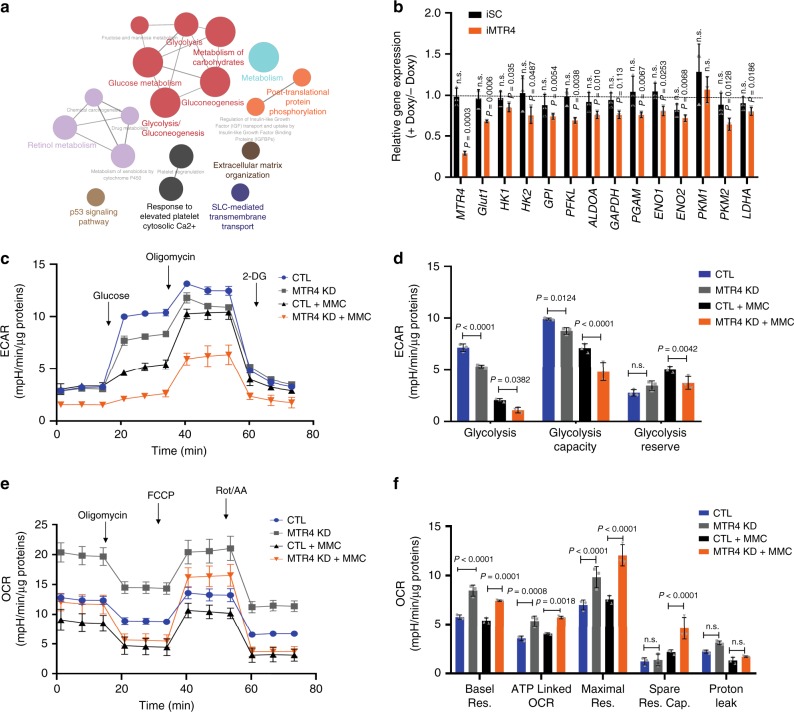
MTR4 is required for glycolysis of HCC cells. **a** Global gene expression analysis of iMTR4 cells before and after Doxy treatment identified differential expressed genes (DEGs), which were enriched in various pathways with pathway analysis and visualized as the overlap graph. **b** The mRNA levels of glycolytic genes in iSC and iMTR4 cells before and after Doxy treatment. Two-tailed, paired *t*-test. Data are presented as mean value ± s.d. *p* value is indicated. n.s., non-significant. *n* = 3 biologically independent samples. **c**, **d** Extracellular acidification rate (ECAR) in control cells and MTR4 KD cells with either MMC (5 µg/ml) or DMSO as a solvent control in response to glucose (10 mM), oligomycin (1 μM), and 2-DG (50 mM). *n* = 3 independent experiments. Data are presented as mean value ± s.d., two-way ANOVA with a Tukey’s multiple comparison test. *p* value is indicated. **e**, **f** Oxygen consumption rate (OCR) in control cells and MTR4 KD cells with either MMC or DMSO as a solvent control in response to oligomycin (1 μM), fluorocarbonyl cyanide phenylhydrazone (FCCP, 1.5 μM), rotenone/antimycin A (Rot/AA, 0.5 μM). *n* = 3 independent experiments for each group. Data are presented as mean value ± s.d., two-way ANOVA with Tukey’s multiple comparison test. *p* value is indicated. Res., respiration; Cap, capability. Source data are provided as a Source Data file.

To reveal the mechanism underlying MTR4-dependent tumorigenesis of HCC cells, we performed RNA-seq analysis of HCC cells before and after the inducible knockdown of MTR4 to identify the genes that are affected by MTR4. Over 1249 genes were differentially expressed (fold change > 2.0) between isogenic control and HCC cells with MTR4 inducible knockdown. Based on KEGG-functional annotation and gene ontology (GO) terms, the differentially expressed genes (DEGs) are significantly enriched in metabolic pathways (Supplementary Fig. 3). Using Consensus path DB (CPDB), the DEGs are enriched in signaling and metabolic pathways, especially glycolysis and carbohydrate metabolism^14^ (Fig. 2a). Gene Set Enrichment Analysis (GSEA) further reveals that glycolytic pathway is significantly deregulated in the MTR4-depleted HCC cells (Supplementary Fig. 3). In this context, the expression of several key glycolytic genes such as *GLUT1* and *PKM2* was decreased in HCC cells after MTR4 knockdown (Fig. 2b). Doxycycline did not affect the expression of these glycolytic genes in HCCs that inducibly expressed the scramble shRNA, indicating that doxycycline at the used dosage alone did not affect the expression of the metabolic genes (Fig. 2b). Therefore, MTR4 is important to maintain the expression of glycolytic genes in HCC cells.

**Fig. 3:**
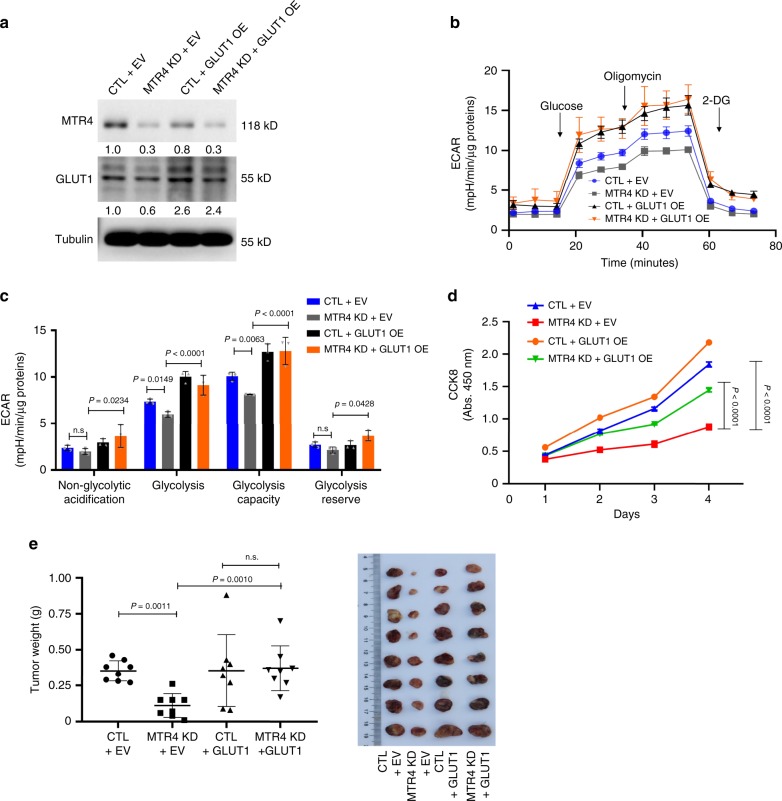
Ectopic expression of GLUT1 rescued the defective tumorigenesis of HCC cells after MTR4 silencing. **a** Ectopic expression of GLUT1 in control and MTR4 KD cells was analyzed for MTR4 and GLUT1 by western blotting. Representative data from two independent experiments are shown. **b**, **c** ECAR in control cells and MTR4 KD cells transfected with either empty vector (EV) or plasmids expressing GLUT1 (GLUT1 OE) in response to glucose, oligomycin, and 2-DG. Data are presented as mean value ± s.d., two-way ANOVA with a Tukey’s multiple comparison test. *n* = 3 independent experiments for each group. **d** The proliferation of indicated cells were analyzed with CCK8 assay. Data are presented as mean value ± s.d. Two-way ANOVA with a Tukey’s multiple comparison test. *n* = 3 independent biological samples. **e** The weight of tumors formed by indicated cells in NSG mice was measured and compared. Data are presented as mean value ± s.d. One-way ANOVA, followed by Bonferroni post-tests. *n* = 8 independent biological samples for each group. *p* value is indicated. Source data are provided as a Source Data file.

Since MTR4 knockdown decreases the proliferation of HCC cells, to evaluate the impact of MTR4 knockdown on metabolism, we synchronized the proliferation status of MTR4 knockdown and control HCC cells by treating the cells with mitomycin C for 2 h (Supplementary Fig. 4). Consistent with the finding that the MTR4 knockdown reduces the expression of glycolytic genes, the silence of MTR4 expression decreased glycolytic activity and increases oxidative phosphorylation, indicating that MTR4 drives the metabolic shift from oxidative phosphorylation to glycolysis (Fig. 2c–f). The knockdown of MTR4 reduced the expression of the key glycolytic genes such as *GLUT1* and *PKM2* (Fig. 3a, b). To understand the mechanism underlying MTR4-driven glycolysis and tumorigenesis, we tested the hypothesis that the MTR4-dependent expression of glycolytic genes is important for mediating MTR4-driven tumorigenesis. Considering the importance of GLUT1 in cancer metabolism, we rescued the expression levels of GLUT1 in the MTR4-silenced HCC cells through ectopic expression of *GLUT1* gene. The restoration of GLUT1 protein levels in MTR4-silenced HCC cells partially rescued the defects in glycolysis and cellular proliferation in vitro as well as tumor growth in vivo (Fig. 3b–e, Supplementary Fig. 5). Therefore, MTR4-dependent expression of glycolytic genes plays important roles in driving cancer metabolism and tumorigenesis of HCC.

### MTR4 regulates the alternative splicing (AS) in HCC cells

**Fig. 4:**
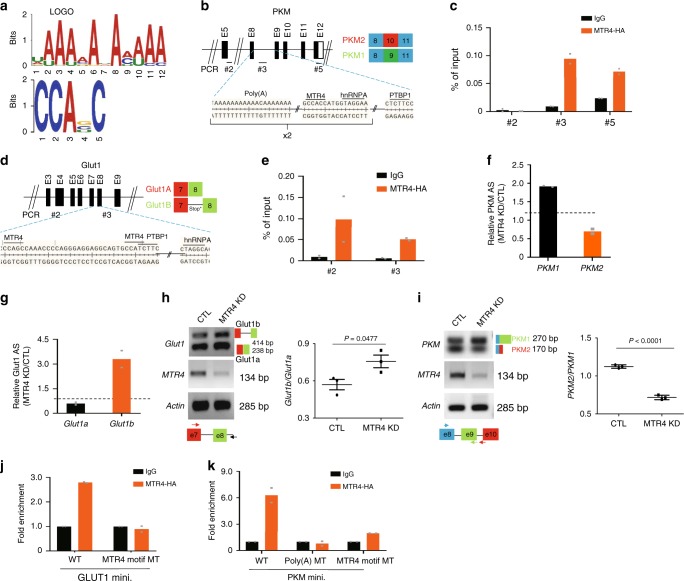
MTR4 binds to a large panel of pre-mRNA and regulates the alternative splicing of *GLUT1* and *PKM* genes. **a** MTR4 binding motifs, UAAAAAA(U)AA(C)A(U)AA (which is the conventional MTR4 binding region, poly(A) sequences) and CCAG(C/U/G)C, are defined by analyzing sequence of 3301 peaks from RIP-seq with motif analysis tool, MEME-Chip. **b**, **d** A schematic of *PKM* gene (**b**) or *GLUT1* gene (**d**) displaying potential binding motifs for MTR4 and two RNA alternative splicing factors PTBP1 and hnRNPA. Numbers indicate the locations of primers for PCR. **c**, **e** RIP analysis of the binding of MTR4 to the predicted MTR4 binding motifs of *PKM* gene (**c**) or *GLUT1* gene. **e** Due to the lack of anti-MTR4 antibodies that can be used for immunoprecipitation, PLC/PRF5 cells expressing HA-tagged MTR4 were subjected to immunoprecipitated with anti-HA antibody or IgG. Eluted RNA was analyzed by RT-qPCR and display as % of input. *n* = 2 independent experiments. Data are presented as mean values. Numbers indicate the locations of primers for PCR (**f**, **g**) Relative mRNA levels of *PKM2/PKM1* (**f**) or *GLUT1b/GLUT1a* (**g**) in MTR4 KD cells versus control cells. Dotted line indicates 1. Data are presented as mean values. *n* = 2 independent experiments. **h**, **i** Alternative splicing (AS) of minigenes (mini.) of *PKM* (**h**) or *GLUT1* (**i**) genomic DNA containing exons undergoing AS in MTR4 KD or control cells. Primers for PCR analysis are indicated by arrows. Data are presented as mean value ± s.d. Two-tailed, unpaired *t*-test. *p* value is indicated. *n* = 3 independent experiments. **j**, **k** RIP analysis of MTR4 binding to the WT and MTR4 binding motif mutant *PKM* minigene (**j**) or *GLUT1* minigene (**k**). PLC/PRF5 cells expressing HA-tagged MTR4 in combination with vector expressing indicated minigenes were subjected to immunoprecipitation with anti-HA antibody or IgG. Eluted RNA was analyzed by RT-qPCR and display as fold changes over IgG. Data are presented as mean values. *n* = 2 independent experiments. Source data are provided as a Source Data file.

To understand how MTR4 maintains the expression of glycolytic genes, we performed RNA immunoprecipitation followed by sequencing (RIP-seq) to identify the pre-mRNAs bound by MTR4 in HCC cells. MTR4 binds to the intronic region of half of the bound transcripts (1619/3301), suggesting that pre-mRNA is the primary target bound by MTR4 (Supplementary Fig. 6a). Consistent with the RNA-seq data, KEGG-functional annotation analysis reveals that the transcripts bound by MTR4 are significantly enriched in metabolism and cell cycle pathways (Supplementary Fig. 6b, c). Using motif analysis tools such as MEME-Chip to screen for putative MTR4 binding motifs^15^, two MTR4 binding motifs have been identified: (UAAAAAA(U)AA(C)A(U)AA), the expected motif considering that MTR4 is a helicase unwinding poly-A tail sequence^9^, and motif CCAG(C)(U)C that is found in intronic region of pre-mRNA (Fig. 4a).

While previous studies have shown that MTR4 plays an important role in RNA surveillance and RNA stability^8,16^, we hypothesized that MTR4 might also regulate mRNA levels by regulating the alternative splicing of pre-mRNAs. To test this hypothesis, we analyzed the global mRNA splicing in HCC cells before and after MTR4 depletion. We identified differential alternative splicing (AS) events between the two sample groups (KD vs Control) corresponding to all five basic types of AS patterns, resulting with 1713 skipped exon (SE), 131 alternative 5′ splice site (A5SS), 192 alternative 3′ splice site (A3SS), 307 mutually exclusive exons (MXE), and 103 retained intron (RI) differential AS events (Supplementary Fig. 6f). We used DAVID (https://david.ncifcrf.gov/home.jsp, version 6.8) to perform REACTOME pathway analysis for the identified genes, indicating that the differential AS genes are enriched in the metabolic pathways (Supplementary Fig. 6c). To evaluate the potential significance of MTR4 in inducing the abnormal AS events in HCC samples from patients, we compared the potential target genes of MTR4 with the genes undergoing abnormal AS in HCC cells after MTR4 KD. Our analysis indicates significant overlap between the predicted MTR4 target genes with the genes undergoing change in AS in HCC cells after MTR4 KD, suggesting that the overexpression of MTR4 in HCCs plays a key role in inducing abnormal AS in HCC of patients^10^ (Supplementary Fig. 6d, e).

### MTR4 regulates AS by recruiting PTBP1 to its target pre-mRNAs

To elucidate the mechanism how MTR4 regulates AS in HCC cells, we focused on the key glycolytic genes *GLUT1* and *PKM2*, both are MTR4 targets as confirmed by the presence of MTR4 binding motifs in their pre-mRNAs and by the standard RIP assays, indicating that MTR4 binds to the intronic regions of pre-mRNAs of these genes (Fig. 4b–e). After *MTR4* knockdown, the AS of the pre-mRNA of these genes is affected so that the levels of mRNA encoding the functional GLUT1 and PKM2 are reduced (Fig. 4f, g). In contrast, the levels of the alternatively spliced mRNA isoforms, *GLUT1b*, a novel alternative spliced *GLUT1* mRNA, or *PKM1*, are increased (Fig. 4f, g).

**Fig. 5:**
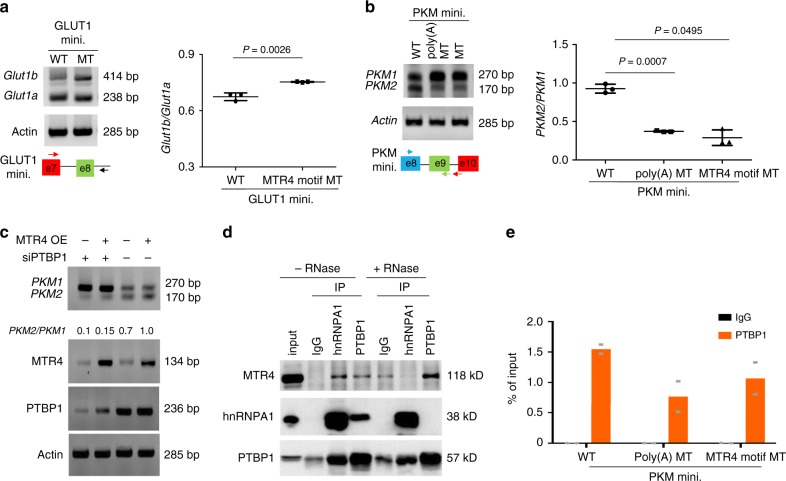
MTR4 regulates AS of its target pre-mRNA by recruiting PTBP1. **a** AS of WT and two MTR4 binding motif mutant (MT) including poly(A) MT in *GLUT1* minigene in PLC/PRF5 cells. Data are presented as mean value ± s.d. Two-tailed, unpaired *t*-test. *p* value is indicated. *n* = 3 independent experiments. **b** AS of WT and two MTR4 binding motif mutant (MT) including poly(A) MT in *PKM* minigene in PLC/PRF5 cells. Data are presented as mean value ± s.d. One-way ANOVA with a Dunnett’s multiple comparison test. *p* value is indicated. n.s., non-significant. *n* = 3 independent experiments. **c** Impact of PTBP1 knockdown on AS of PKM minigene in PLC/PRF/5 cells with or without MTR4 overexpression. The levels of PKM1 and PKM2 isoforms were analyzed using primers described in (**b**). Representative data from two independent experiments are shown. **d** The co-immunoprecipitation analysis of the endogenous MTR4, hnRNPA1, and PTBP1 in PLC/PRF/5 cells in the absence or presence of RNase. The cell lysate was subjected to immunoprecipitation (IP) with indicated antibodies. The presence of various proteins in the immunoprecipitate was examined by western blotting using indicated antibodies. Because IP grade anti-MTR4 antibody is not commercially available, we did not perform the MTR4 IP. Representative data from two independent experiments are shown. **e** RIP analysis of the binding of PTBP1 to the WT and MTR4 binding motif mutant *PKM* minigene in PLC/PRF5 cells. Data are presented as mean values. *n* = 2 independent experiments. Source data are provided as a Source Data file.

To determine whether MTR4 binds to the pre-mRNA via the predicted binding motifs, we constructed minigenes of *GLUT1* and *PKM2* genomic DNA that contains exons flanking the introns with the predicted MTR4 binding sites. Consistent with the data from the endogenous genes, the knockdown of *MTR4* increases the splicing of the pre-mRNA transcribed from the minigenes to the *GLUT1b* and *PKM1* isoforms, indicating that these minigenes could serve as a model to study the mechanisms how MTR4 regulates AS (Fig. 4h, i). In this context, the mutation of the predicted MTR4 binding motifs within the minigenes abolishes the binding of MTR4 to the pre-mRNA transcribed from these minigenes, confirming that MTR4 binds to the predicted binding motifs of the pre-mRNA (Fig. 4j, k). In addition, similarly to the impact of MTR4 knockdown, the mutation of the MTR4 binding motifs within the minigenes led to increased splicing of the pre-mRNAs transcribed from the minigenes to *GLUT1b* and *PKM1* isoforms respectively, indicating that the binding of MTR4 to its target pre-mRNA is required for its function in AS (Fig. 5a, b).

To understand how MTR4 regulates AS events, we examined the functional relationship between MTR4 and PTBP1, which is a key regulator of AS^13^. While the overexpression of MTR4 induced the splicing of pre-mRNA transcribed from *PKM* minigene to *PKM2* isoform in HCC cells, the silence of *PTBP1* in HCC cells inhibits the AS of the pre-mRNA transcribed from *PKM* minigene to *PKM2*, suggesting that PTBP1 is involved in MTR4-dependent splicing events (Fig. 5c). In support of this notion, co-immunoprecipitation experiments indicate that MTR4 interacts with PTBP1 in a RNA-independent manner (Fig. 5d). In contrast, MTR4 interacts with another RNA-binding protein hnRNPA1 in a RNA-dependent manner (Fig. 5d). Therefore, MTR4 might recruit PTBP1 to its target pre-mRNAs through protein–protein interaction. In further support of this notion, the mutation of the MTR4 binding motifs within the *PKM* minigene significantly reduces the binding of PTBP1 to the pre-mRNA transcribed from the minigene (Fig. 5e). In summary, these data demonstrate that MTR4 regulates AS of its target genes by recruiting PTBP1 to the target pre-mRNAs.

### c-Myc directly activates the transcription of MTR4 in cancer cells

**Fig. 6:**
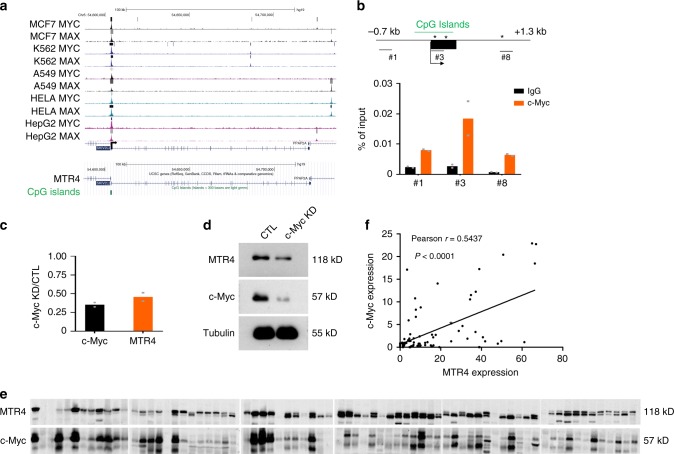
MTR4 is a direct transcriptional target of c-Myc. **a** The analysis of Chip-seq data of c-Myc in human cancer cell lines available in the ENCODE database predicted that c-Myc binds to the promoter region of MTR4. **b** ChIP analysis of the binding of c-Myc to the predicted c-Myc binding sites within the MTR4 promoter. A schematic of MTR4 regulatory region (−0.7 to +1.3 kb from TSS) was shown. Arrow and black rectangle represent the transcriptional start site (TSS) and exon1, respectively. * indicates the canonical E box. Green bar indicates CpG Islands. Numbers indicates the regions for PCR. Chromatin DNA fragments were immunoprecipitated with anti-c-Myc antibody or IgG. Data are presented as mean values. *n* = 2 independent experiments. **c** The mRNA levels of *c-Myc* and *MTR4* were detected by q-RT-PCR and normalized by *actin*. Data are presented as mean values. *n* = 2 biologically independent samples. **d** The protein levels of c-Myc and MTR4 after the knockdown of c-Myc in HCC cells. Representative data from two independent experiments are shown. **e**, **f** The protein levels of c-Myc and MTR4 in HCC patient samples were analyzed by western blotting (**e**) and their correlation analyzed (**f**). The protein samples were run on multiple gels with one common sample run on all gels as a quantitative control. Two-tailed Pearson correlation test. *p* value and pearson’s correlation coefficient *r* are indicated. *n* = 68 HCC patient samples. Source data are provided as a Source Data file.

To further understand the importance of MTR4 pathway in tumorigenesis of HCC, we searched for upstream regulators that might activate MTR4 pathway. Because the critical oncoprotein c-Myc promotes glycolysis partly by regulating AS of key glycolytic genes such as PKM2^12,17^, we examined the functional interaction between c-Myc and MTR4. The analysis of the chromatin immunoprecipitation-sequencing (ChIP-seq) data of c-Myc in several cancer cells in the ENCODE database reveals that c-Myc binds to the promoter of the MTR4 gene (Fig. 6a). This prediction is confirmed by CHIP analysis, identifying a 695 bp CpG Isalnds around TSS and two non-canonical E boxes (CACGCG, CACGAG) that could be bound by c-Myc (Fig. 6b). The knockdown of c-Myc in HCC cells reduces the expression of both mRNA and protein level of MTR4, indicating that c-Myc directly activates the expression of MTR4 (Fig. 6c, d). The physiological relevance of this finding is further supported by the findings that the protein levels of c-Myc are positively correlated with the levels of MTR4 in HCC tissues from patients (Fig. 6e, f). In summary, MTR4 is a key downstream mediator of c-Myc in promoting cancer metabolism of HCC.

## Discussion

Both genetic and epigenetic abnormalities of cancer cells contribute to cancer progression and drug resistance. Accumulating data have indicated that cancer cells develop aberrant AS profile that significantly increases the complexity of the oncogenic network to promote tumorigenesis, but at the same time, provide potential cancer-specific therapeutic targets^11^. Therefore, it is critical to identify oncogenic pathways that induce the abnormal AS events in human cancers. We discovered that the RNA helicase MTR4 is required for the tumorigenesis of HCC by maintaining the expression of the key glycolytic genes in HCC cells. MTR4 accomplishes this metabolic role by regulating AS of a large panel of pre-mRNAs, including the ones transcribed from the key glycolytic genes. Because MTR4 is overexpressed in a few types of human cancers including HCC, and the target pre-mRNAs of MTR4 are significantly overlapped with the pre-mRNAs undergoing abnormal AS in HCC patient samples^10^, MTR4 could be a master regulator of the oncogenic AS events in HCC. Together with our finding that the acute depletion of MTR4 in established HCC abolishes the tumor growth in vivo, we propose that MTR4 could represent an effective and specific therapeutic target for treating HCC.

As a RNA helicase, extensive studies have been devoted to elucidate the biochemical roles of MTR4 in RNA processing, but the roles of MTR4 in physiology and tumorigenesis remain poorly understood. Previous studies have shown that the primary roles of MTR4 include the binding to the 3′ UTR of a subset of RNA such as viral RNA and target them for degradation, and thus are involved in regulating RNA stability^18^. Our findings reveal a novel role of MTR4 in AS, a highly regulated RNA processing activity important for tumorigenesis. In this context, MTR4 regulates AS by binding to the intronic region of pre-mRNAs and recruiting PTBP1 to its target pre-mRNA through protein–protein interaction. The pre-mRNAs targeted by MTR4 are enriched in metabolic and cell cycle pathways, indicating that MTR4 has diversified and broad physiological and oncogenic functions. In support of this notion, we demonstrate that MTR4-dependent expression of glycolytic genes is important for glycolysis and tumorigenesis of HCC cells, and MTR4 is an independent prognostic marker for HCC. In this context, cancer cells also develop alternative mechanisms to increase the expression of glycolytic genes such as GLUT1 by epigenetics and to maintain glycolysis by inducing PUMA-dependent suppression of oxidative phosphorylation^4,19^.

The powerful and ubiquitous oncoprotein c-Myc is known to promote cancer metabolic switch from oxidative phosphorylation to glycolysis via multiple mechanisms such as the regulation of AS of the key glycolytic genes including *PKM2*^17^. It has been reported that c-Myc induces the expression of PTBP1, hnRNPA1, and hnRNPA2, resulting in exon 10 inclusion and PKM2 production^12^. Our findings demonstrate that c-Myc directly activates the expression of MTR4 in HCC cells and thus provide an alternative mechanism how c-Myc regulates AS of glycolytic genes in HCC in order to induce cancer metabolism. Considering that c-Myc is overexpressed in a majority of human cancers and promotes their tumorigenesis, but MTR4 is overexpressed in limited types of human cancers, we predict that c-Myc is necessary but not sufficient for activating the transcription of MTR4, and other HCC-specific transcriptional factors are required to collaborate with c-Myc in activating MTR4 transcription in HCC.

## Methods

### Patients and tissue samples

After obtaining adequate informed consent, hepatocellular carcinoma (HCC) tissue and adjacent normal tissue (ANT, exceeding the edge of the tumor by at least 2 cm) were obtained from 77 patients who underwent curative resection for HCCs at Nanfang Hospital of Southern Medical University, Guangzhou, China, between November 2010 and May 2015. Patients were regularly assessed for survival analysis via outpatient follow-up or telephone interviews. All patients satisfied the following inclusion criteria: they had not received any other treatments before this surgery; the surgical margins were confirmed to contain no residual carcinoma tissue; Clinicopathological information on age, gender, tumor size, tumor number, tumor capsule, differentiation, AFP, Cirrhosis, MVI (Micro-vascular), PVTT (invasion portal vein tumor thrombosis), TNM stage, and BCLC stage was available. This study was approved by IRB of Nanfang Hospital at Southern Medical University and was performed according to the Declaration of Helsinki (6th revision, 2008).

All patient samples were immediately frozen in liquid nitrogen and stored at −80 °C until the extraction of RNA. For the correlation study of mRNA expression levels and survival time, total RNA was extracted from tissues using TRIzol reagent (Invitrogen, Carlsbad, CA) according to the manufacturer’s instructions. cDNA was synthesized using PrimeScript cDNA kit (Takara Bio, Kusatsu, Shiga, Japan). Quantitative real-time PCR was performed using an SYBR Premix Ex Taq (Takara Bio). 18S RNA levels were used as the internal control. The PCR primers were listed in Supplementary Table 1. The correlation between the gene expression and clinicopathological variables, including age, gender, tumor size, tumor number, tumor capsule, differentiation, AFP, Cirrhosis, MVI, PVTT, TNM stage, and BCLC stage was evaluated with the Chi-square test. Patients were divided into two groups based on the *MTR4* levels: patients with the top 25 percentile of the highest MTR4 expression in HCC and rest of the patients. The postoperative survival rate was analyzed with the Kaplan–Meier method, and the difference in survival was assessed with the log-rank test. Univariate and multivariate analyses were based on the Cox proportional hazards regression model. All the statistical analyses were performed with IBM SPSS Statistics 20.0 (IBM, IL, USA). Two-sided *p* values were calculated, and *p* < 0.05 was considered a statistically significant result.

### Cell lines, viral vectors, constructs, and transfection

293 FT cell line was purchased from Thermo Fisher Scientific (Waltham, MA). Other cell lines were obtained from ATCC (American Type Culture Collection, USA) and maintained in our laboratory. All cell lines were cultured at 37 °C with 5% CO_2_ in DMEM (Invitrogen, Carlsbad, CA) containing 10% fetal bovine serum (FBS) (Gibco, Australia). To establish cells with stably silenced gene, lenti-viruses expressing short hairpin RNA (shRNA) by the pLKO.1 puro vector were used (Addgene #8453) according to Addgene’s pLKO.1 protocol. *MTR4* shRNA target sequence is GGAAGGATTTCCGATGGATTT. shRNA oligomers were designed, annealed, and inserted into PLKO.1 puro according to Addgene’s pLKO.1 protocol. Lenti-viruses were produced by co-transfecting the pLKO.1 vectors, packaging plasmid psPAX2 (Addgene plasmid 12260, Addgene, Cambridge, MA) and envelope plasmid pMD2.G (Addgene plasmid 12259) into HEK293FT cells. For the doxycycline (Doxy)-inducible MTR4 knockdown, tet-pLKO-puro vector (Addgene #21915) and Fuw-M2rtTA (Addgene 20342) were used. Seventy-two hours post infection, cell debris in the media was removed by centrifugation at 2000 rpm for 10 min and lenti-virus concentrated by Lenti-X concentrator (Clonetech, Mountain View, CA). One day before the viral infection, cells were plated into a six-well plate at a density of 1 × 10^5^ cells/well and transduced in the presence of 5 μg/ml polybrene for 72 h. The cells were selected for puromycin resistance (2 μg/ml) for 7 days. To overexpress Glut1, the full length of *Glut1* cDNA was inserted into pLenti-CMV GFP vector (Addgene 17448) linearized by Xba1 digestion.

### AS analysis using minigene

Due to technical issues, *PKM* minigene was constructed by combining two fragments between intron 8 and intron 11 were subcloned into the pLenti-CMV plasmid after PCR amplification using H1/H9 gDNA as templates. All plasmid constructed in each step was confirmed by sequencing. First fragments (3654 bp) were obtained by PCR using one primer pair hPKM-in8-F01 and hPKM-R02 and modified for Gibson assembly by second round PCR using one pair of primers hPKM-in8-gF and hPKM-R02-g. Modified fragments were applied to Gibson assemble (NEBuilder HiFi DNA Assembly Master Mix) with vector backbone plasmid pLenti-CMV GFP vector (Addgene 17448) linearized and by BamHl/Sall digestion. Second fragments were obtained by PCR using the primer pair hPKM-F02 and hPKM-in11-R, modified by PCR using another primer pair hPKM-Frag2-gF2 and hPKM-gR, and applied to Gibson assembly with pLenti-CMV vector including first fragment, which was prepared by Sall digestion and rSAP-mediated dephosphorylation. Point mutations in MTR4 binding motifs were introduced into minigenes by site-directed mutagenesis. For mutations in poly-A tails, two primer pairs (PKM-m1766-F, PKM-m1766-R; PKM-m1906-F, PKM-m1906-R), were used to amplify cDNA products, which were incubated with methylation-dependent endonuclease Dpnl to remove parent template. For mutating the short MTR4 binding motif, another two primer pairs (PKM-m1708-F, PKM-m1708-R; PKM-m1906-F, PKM-m1906-R) were used.

*Glut1* minigene was generated by PCR using the primer pair specific for intron 2 to intron 8 (hGlut1-in2-F, hGlut1-in8-R). Resulting products were modified for Gibson assembly by the second round PCR using the pair of primers hGlut1-in2-gF and hGlut1-in8-gR, and applied to Gibson assembly. Primers for site-directed mutagenesis in the *GLUT1* minigenes were Glut1m-F and Glut1m-R. For analysis of alternative splicing in *PKM* and *GLUT1* genes, PLC/PRF/5 cells were transfected with plasmids expressing the indicated minigenes. Forty-eight hours later, RNA was extracted, reversely transcribed, and applied to PCR detection for alternatively spliced forms. To distinguish minigene-derived transcripts from the endogenous transcripts, reverse primers specific for plasmid backbone were used. Sequence of all primers used in minigene analysis was provided in Supplementary Table 2.

### Cell growth assay

For clonogenic assay, single-cell suspension was prepared by trypsinization and the indicated cell lines seeded onto 6-well plates with a density of 500 cells per well. After culturing for 2 weeks, colonies were stained with crystal violet and counted. Cell Counting Kit 8 (CCK8) assay was performed using 96-well plates. Briefly, cells were seeded at a density of 3–5 × 10^3^ cells/well, and cultured for 24–96 h. CCK-8 solution was added to each well and incubated for 60 min, the plates were measured at 450 nm with a microplate reader.

### Animal experiments

All animal work was approved by Institutional Animal Care and Use Committee (IACUC) of Southern Medical University and University of California, San Diego. To analyze tumor growth in NODSCID mice, cells were harvested, resuspended in 0.2 ml serum-free DMEM mixed with Matrigel at an 1:1 ratio, and injected subcutaneously into the left and right flank of NODSCID mice (5 × 10^6^ cells each). To evaluate the impact of MTR4 depletion on tumorigenicity, mice bearing established tumors about 0.5 cm in diameter were randomized into two groups. Subsequently, Doxy (20 mg/kg body weight) was injected intraperitoneally (i.p.) every day. The control group was injected with the same volume of PBS. In addition, doxy-treated group was continuously provided with doxy containing drinking water (2 mg/L).

### Extracellular acidification rate (ECAR)

Glycolysis Stress Test (103020-100, Agilent, Santa Clara, CA) was performed with the Seahorse XFe96 Extracellular Flux Analyzer (Agilent, Santa Clara, CA). In total, 2 × 10^4^ cells were plated onto wells of a XF96 Cell Culture Microplate (102416-100, Agilent, Santa Clara, CA) and incubated overnight. Plates were equilibrated in unbuffered XF assay medium supplemented with 2 mM glutamine in the absence of CO_2_ for 1 h. Extracellular acidification rates (ECAR) were assayed by the serial addition of Glucose (10 mM), oligomycin (1 μM), and 2-deoxy-glucose (2-DG, 50 mM) to establish glycolysis (ECAR in response to glucose − ECAR before glucose injection), glycolytic capacity (ECAR in response to oligomycin − ECAR before glucose injection), glycolytic reserve (glycolytic capacity − glycolysis), and non-glycolytic acidification (ECAR before glucose injection). Each plotted value is the mean of at least 3 triplicate wells, and normalized to baseline ECAR and total protein levels. Data were presented as mean ± SD, and significance calculated by two-way ANOVA with Tukey’s multiple comparison test.

### Oxygen consumption rate (OCR)

Cell Mito Stress Test (103015-100, Agilent, Santa Clara, CA) was measured with the Seahorse XFe96 Extracellular Flux Analyzer (Agilent, Santa Clara, CA). In total, 2 × 10^4^ cells were plated onto each well of a XF96 Cell Culture Microplate (102416-100, Agilent, Santa Clara, CA) and incubated overnight. The plates were equilibrated in unbuffered XF assay medium supplemented with 10 mM glucose and 2 mM glutamine in the absence of CO_2_ for 1 h. Oxygen consumption rates (OCR) were interrogated by a serial addition of oligomycin (1 μM), carbonyl cyanide 4‐(trifluoromethoxy) phenylhydrazone (FCCP, 1.5 μM) and rotenone/antimycin A (Rot/AA, 0.5 μM) to establish basal respiration (OCR before oligomycin injection − OCR in response to Rot/AA), ATP production (OCR before oligomycin injection −  OCR in response to oligomycin), maximal respiration (OCR in response to FCCP − OCR in response to Rot/AA), spare capacity (maximal respiration − basal respiration), and proton leak (OCR in response to oligomycin − OCR in response to Rot/AA). Each plotted value is the mean of at least three triplicate wells and normalized to baseline OCR and total protein levels. Data are presented as mean ± SD, and significance calculated by two-way ANOVA with Tukey’s multiple comparison test.

### Western blotting and co-immunoprecipitation

Samples were separated on 8–15% SDS PAGE and transferred to nitrocellulose membranes. The blots were incubated in blocking buffer (5% skim milk in PBS with 0.05% Tween 20) with primary antibodies. After washing three times with blocking buffer, the blots were probed with a horseradish peroxidase-conjugated secondary antibody and developed with Supersignal West Pico or Dura (Thermo Fisher Scientific). The following antibodies were used: rabbit polyclonal anti-Myc antibody (13987; Cell Signaling), rabbit polyclonal anti-MTR4 antibody (ab70551; Abcam), anti-Glut1 antibody (ab150299; Abcam), mouse monoclonal anti-α-tubulin antibody (T5168; Sigma-Aldrich), anti-rabbit IgG, HRP-linked antibody (7074S; Cell Signaling), anti-mouse IgG antibody, HRP-linked antibody (7076S; Cell Signaling). For co-immunoprecipitation (Co-IP), cell lysates were prepared in IP lysis buffer (87787, Thermo) with Halt Protease and Phosphatase Inhibitor Cocktail (78430, Thermo) on ice for 30 min. After centrifugation at 12,000 × *g* for 10 min at 4 °C, supernatants were immunoprecipitated with either anti-hnRNPA1 (03-204, Millipore) or anti-PTBP1 (MABE-986, Millipore) antibodies followed with magnetic protein A/G beads (Pierce) for 2 h at 4 °C. The intensity of protein bands was quantified using Image Lab (BioRad). All uncropped and unprocessed scans of western blots are provided as Supplementary Fig. 7 in the Supplementary Information.

### Quantitative PCR analysis

RNeasy Mini Kit (Invitrogen) was used to extract total RNA from cells according to the manufacturer’s instructions. Cells were collected directly into RLT lysis buffer and homogenized using QIAshredder (Qiagen) according to manufacturer’s instructions. RNA was purified with RNeasy Mini kit columns, and genomic DNA removed with RNase-free DNase Kit (Qiagen). TRIzol reagent (Thermo Scientific) was used for RNA extraction from human patient tissues. 0.5 mg of liver tissues was homogenized in 1 ml TRIzol reagent with electric homogenizer and mixed with 0.2 ml chloroform vigorously (Sigma-Aldrich). After incubating at room temperature for 3 min, the mixture was centrifuged at 10,000 × *g* for 15 min. RNA fraction was mixed with the same volume of 100% ethanol and purified with RNeasy column. cDNA synthesis from total RNA was performed using High Capacity cDNA Reverse Transcriptase Kit (Applied Biosystems) following the manufacturer’s instructions. Real-time qPCR was performed with an ABI Prism 7000 (Applied Biosystems) using FastStart Universal SYBR Green Master (Takara). PCR conditions were following: 2 min at 95 °C, 40 cycles of 5 s at 95 °C, and 34 s at 60 °C. The average Ct value for each gene was determined from triplicate reactions and normalized with mRNA levels of β-actin or 18 s. Sequence of all primers used in qPCR analysis was provided in Supplementary Table 1.

### RNA immunoprecipitation and sequencing (RIP-seq)

RNA immunoprecipitation (RIP) was carried out with Magna RIP RNA-Binding Protein immunoprecipitation kit (Millipore; USA) according to the manufacturer’s instruction. Cells overexpressing MTR4-HA were lysed by RIP lysis buffer containing protease inhibitor cocktail (Thermo Fisher, USA) and RNase inhibitor (Invitrogen, USA). To obtain the plasmid expressing MTR4-HA, the full length of *MTR4* cDNA was amplified with primers tagged with HA sequence (TACCCATACGATGTTCCAGATTACGCT) and inserted into pcDNA3 vector that was linearized with Kpnl/Notl digestion. Nuclear pellets were collected and lysed by sonication. Nuclear lysates were incubated with anti-HA antibody or control IgG overnight at 4 °C, followed by incubation with protein A/G Dynabeads (Thermo Fisher, USA) for 2 h. After vigorous washing, pellets were resuspended in TRIzol (Invitrogen, USA). The immunoprecipitated RNAs were purified by RNAeasy Mini Kit (Invitrogen), reversely transcribed, and sequenced. All sequenced reads were checked with FasX-Toolkit and the adapters removed with cutadapter. The processed reads were mapped to hg19 whole genome using TopHat v2.0.13. Peak calling was performed with RIPSeeker. The peak annotation was done with Homer, and the motifs bound by MTR4 identified using MEME-ChIP. To evaluate the impact of MTR4 binding motif on the interaction between RNAs of glycolytic genes and RNA-binding proteins, cDNA derived from cells expressing either WT minigenes or minigenes with mutations in MTR4 binding motifs were applied to PCR using a reverse primer specific for the minigene vector. Sequence of all primers used in RIP-seq analysis was provided in Supplementary Table 3.

### Immunohistochemistry (IHC) analysis of tumor samples

Tumor samples were fixed with 4% paraformaldehyde in 0.1 M phosphate buffer for 24 h, embedded in paraffin, sliced into 4-μm sections and mounted onto glass slides. After dewaxing, the slides were treated with 3% hydrogen peroxide in methanol and blocked with a biotin-blocking kit (DAKO, Germany), incubated overnight with Ki67 monoclonal antibody (1:50, BD Biosciences, USA) in a moist chamber at 4 °C. After washing three times with PBS, the slides were incubated with biotinylated goat anti-mouse antibodies for 1 h. The slides were stained with DAKO liquid 3,3′-diaminobenzidine tetrahydrochloride (DAB), followed by their counterstaining with Mayer’s hematoxylin and examined under a microscope. The protein level of Ki67 was determined by semi-quantitative IHC detection.

### Cell cycle analysis

Cells were seeded onto 6-well plates at a density of 1 × 10^6^ cells/well and treated with MMC (5 µg/ml) for 12 h. Cells were fixed with cold 70% ethanol for 24 h at 4 °C and incubated with 0.5 mg/ml of propidium iodide (PI) along with 0.1 mg/ml of RNase A (MultiSicences, China). Cell cycle analysis was performed using a flow cytometry (FACScan, BD Biosciences), and analyzed with the ModFIt LTV4.1.7 software (BD Biosciences). Cell debris and dead cells were gated out for cell cycle analysis.

### Chromatin immunoprecipitation (ChIP)

After protein was cross-linked to DNA with 1% formaldehyde, the nuclei were extracted and digested with Micrococcal Nuclease to give rise to DNA fragments of ~150–600 bp. After immunoprecipitation with anti-c-Myc antibody overnight at 4 °C, the crosslinking of the immunoprecipitated chromatin was reversed, and the amount of DNA in the immunoprecipitate measured by qPCR.

### RNA-seq analysis of alternatively spliced mRNA isoforms

We mapped RNA-seq reads to the human genome (H. sapiens, GRCh38) and transcriptome (Ensembl, release 84) using the software hisat2 (v2.1.0) with the default parameters. The differential alternative splicing (AS) events between the two sample groups (KD vs Control) were analyzed corresponding to all five basic types of AS patterns using rMATS (rMATS). We ran rMATS with -c 0.0001 parameter and then detected significant splicing events using a cutoff at FDR < 5% and |IncLevelDifference| ≥5%, resulting in 1713 SE, 131 A5SS, 192 A3SS, 307 MXE, and 103 RI differential AS events.

### Quantification and statistic analysis

The statistical significance of Kaplan–Meier survival plot was determined by log-rank analysis. Univariate and multivariate analyses were based on the Cox proportional hazards regression model. Statistical significance in tumor growth rates was tested by repeated measure ANOVA. To compare the tumor weights between two groups, non-parametric Mann–Whitney test was used. For other experiments, significant difference was determined by *t*-test and ANOVA. Asterisks indicate significant difference: ns, not significant. We assumed normal distribution for the data having a small number. All statistical analyses of patient’s survival were performed with IBM SPSS Statistics 20.0 (IBM). Other analyses were performed with PRISM. *N* was indicated in the figure legends. No statistic method was used for determining sample size, blinding and randomizing.

### Biological materials availability

All biological materials can be obtained by sending the request to the corresponding authors.

### Reporting summary

Further information on research design is available in the Nature Research Reporting Summary linked to this article.

## Supplementary information

Supplementary Data 1–7

Reporting Summary

## Data Availability

The RNA-seq data in Fig. 2a and Supplementary Fig. 3 are available in the NCBI Gene Expression Omnibus (GEO) under the accession number GSE129263). The RIP-seq data in Fig. 6a and Supplementary Fig. 5 are available in the NCBI Gene Expression Omnibus (GEO) under GSE129261. The transcriptional dataset on 225 HCC tissues and 220 non-cancerous liver tissues were obtained from Gene Expression Omnibus (GEO) database under the accession number GSE14520. The images of uncropped and unprocessed western blot and gels are included in the Supplementary Fig. 7. Source data underlying Figs. 1c-f, 1h-i, 1k-l, Fig. 2b-f, Fig. 3b-e, Figs. 4c, 4e, 4h-k, Fig. 5a-b, Fig. 6b, Supplementary Fig. 1, Supplementary Fig. 2b, c, e-h, k, Supplementary Fig. 5a-c, are provided as a Source Data file.

## Code availability

No custom scripts were used in the data analysis.
