# Supplementary material for: MTR4 drives liver tumorigenesis by promoting cancer metabolic switch through alternative splicing
Source: Nat Commun. 2020 Feb 5;11:708. doi: 10.1038/s41467-020-14437-3 (PMC7002374; doi:10.1038/s41467-020-14437-3)
Supplement: Supplementary file 1 — Supplementary Data 1–7 [file 41467_2020_14437_MOESM1_ESM.pdf]

Supplementary Information

Title: MTR4 drives liver tumorigenesis by promoting cancer metabolic switch through alternative splicing

Yu et al.

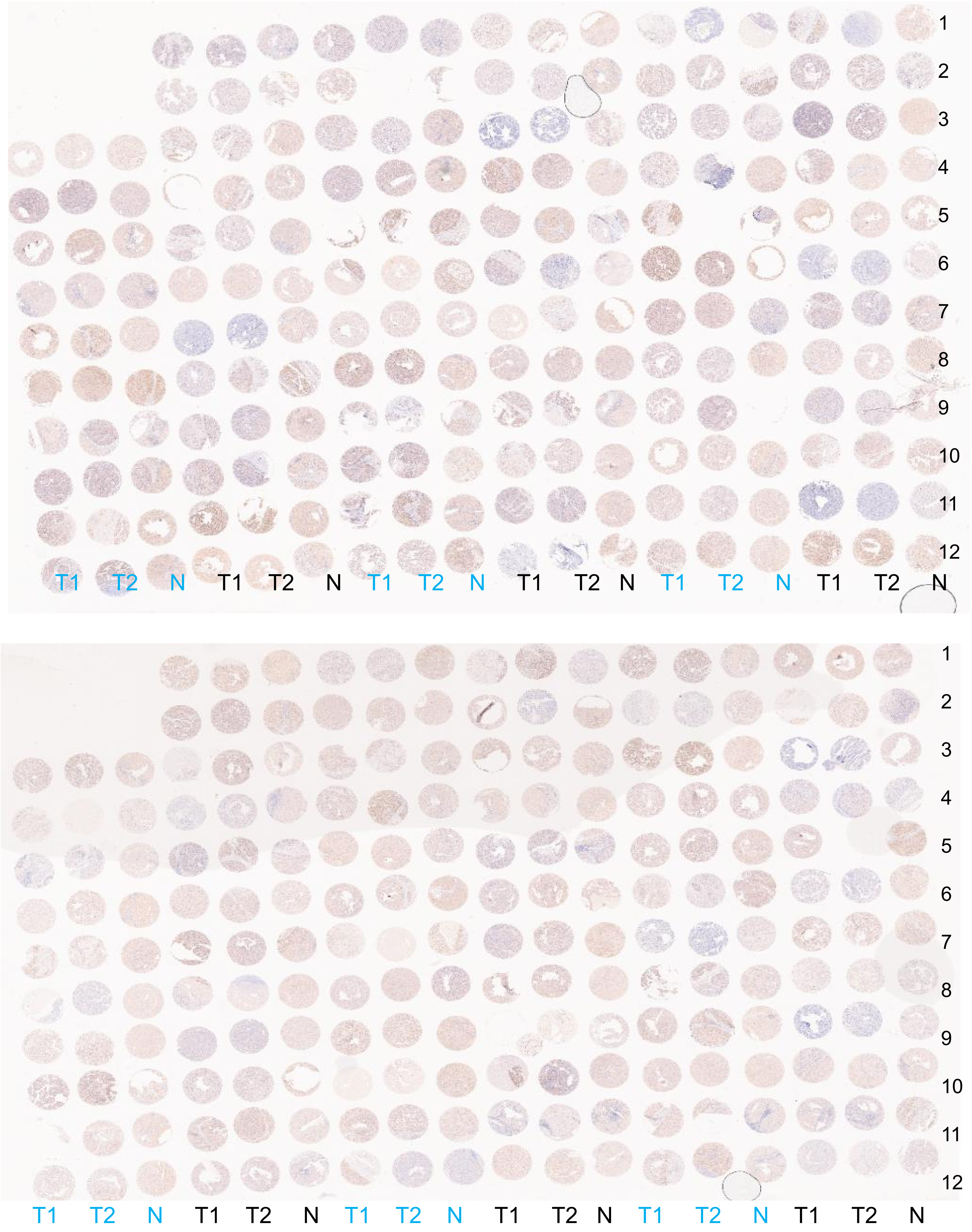

Supplementary Figure 1. MTR4 was overexpressed in HCC tissues. Scan images of IHC analyses of HCC samples on tissue chips for scoring. For each patient, one normal adjacent tissue (N), and two tumor tissues (T1 and T2) were included.

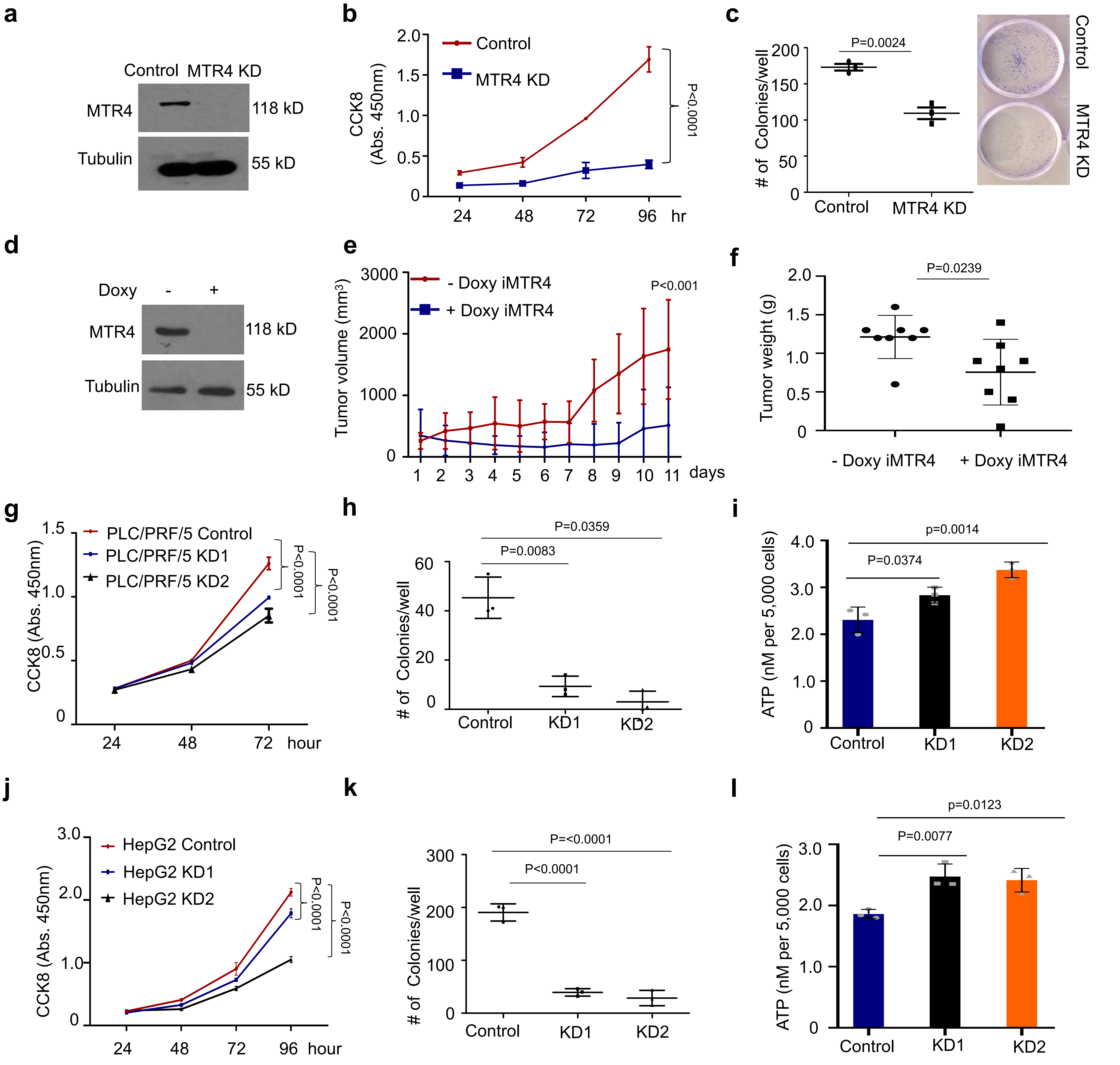
a
Control MTR4 KD
MTR4
118 kD
Tubulin
55 kD
b
Control
MTR4 KD
CCK8 (Abs. 450nm)
P<0.0001
24 48 72 96 hr
c
P=0.0024
# of Colonies/well
Control MTR4 KD
Control
MTR4 KD
d
Doxy - +
MTR4
118 kD
Tubulin
55 kD
e
- Doxy iMTR4
+ Doxy iMTR4
P<0.001
Tumor volume (mm³)
days
f
P=0.0239
Tumor weight (g)
- Doxy iMTR4 + Doxy iMTR4
g
PLC/PRF/5 Control
PLC/PRF/5 KD1
PLC/PRF/5 KD2
P<0.0001
P<0.0001
CCK8 (Abs. 450nm)
24 48 72 hour
h
P=0.0359
P=0.0083
# of Colonies/well
Control KD1 KD2
i
p=0.0014
P=0.0374
ATP (nM per 5,000 cells)
Control KD1 KD2
j
HepG2 Control
HepG2 KD1
HepG2 KD2
P<0.0001
P<0.0001
CCK8 (Abs. 450nm)
24 48 72 96 hour
k
P=<0.0001
P<0.0001
# of Colonies/well
Control KD1 KD2
l
p=0.0123
P=0.0077
ATP (nM per 5,000 cells)
Control KD1 KD2

Supplementary Figure 2. MTR4 is required for tumor growth *in vitro* and *in vivo*. (a, b) Validation of knockdown of MTR4 (a) and CCK8 assay (b) in HepG2 cells. HepG2 cells were transduced by lenti-virus expressing either shRNA targeting MTR4 (MTR4 KD) or non-specific control scramble shRNA (Control) and analyzed for cell proliferation. Values represent means ± s.d. n=3 biologically independent samples. 2-way ANOVA, followed by Bonferroni post-tests. p values are indicated. Wester blotting (c) Colony formation assay in the control cells and MTR4 KD cells. Representative images showed colonies stained with crystal violet. The number of colonies in each well was counted. n=3 biologically independent sample. Data are presented as mean values ± s.d. Two tailed, unpaired t-test. p values are indicated. (d) Inducible MTR4 knockdown in HepG2 cells (iMTR4 HepG2) was confirmed by Western blotting after the treatment with 1μg/ml doxycycline (Doxy) for 4 days. Representative data from two independent experiments are shown (e, f) The volumes (e) and the weight (f) of tumors formed by iMTR4KD HepG2 in NSG mice were measured after the induction of MTR4 knockdown by daily i.p. injection of doxy (20 mg/kg body weight) for indicated days. Individual tumor volumes were measured every day after doxy treatment. At the end of the treatment, the weight of all tumors in each group was compared. Repeated measures ANOVA, followed by Bonferroni post-tests. n= 8 independent samples for each group. Data are presented as mean values ± s.d. *p* values are indicated. (g, j) CCK8 assay in PLC/PRF/5 cells (g) and HepG2 cells (j). PLC/PRF/5 cells or HepG2 cells were transduced by lenti-virus expressing either two different MTR4 shRNAs (KD1 and KD2) or control scramble shRNA, and analyzed for cell proliferation using CCK8. n=3 biologically independent samples. Data are presented as mean values ± s.d. 2-way ANOVA, followed by Dunnett's multiple comparisons test. p values are indicated. (h, k) Colony forming assay in PLC/PRF/5 cells (h) and HepG2 cells (k) PLC/PRF/5 cells or HepG2 cells transduced by lenti-virus expressing either two different MTR4 shRNAs or scramble shRNA were analyzed for colony formation assay. The number of colonies in each well was counted. n=3 biologically independent experiments. Data are presented as mean values ± s.d. One-way ANOVA, followed by Dunnett's multiple comparisons test. p values indicated. (i, l) ATP measurement in PLC/PRF/5 cells (h) and HepG2 cells (k). PLC/PRF/5 cells or HepG2 cells transduced by lenti-virus expressing either two different MTR4 shRNAs or scramble shRNA were analyzed for total ATP with luminescence-based method. n=3 biologically independent experiments. Data are presented as mean values ± s.d. One-way ANOVA, followed by Dunnett's multiple comparisons test. p values are indicated.

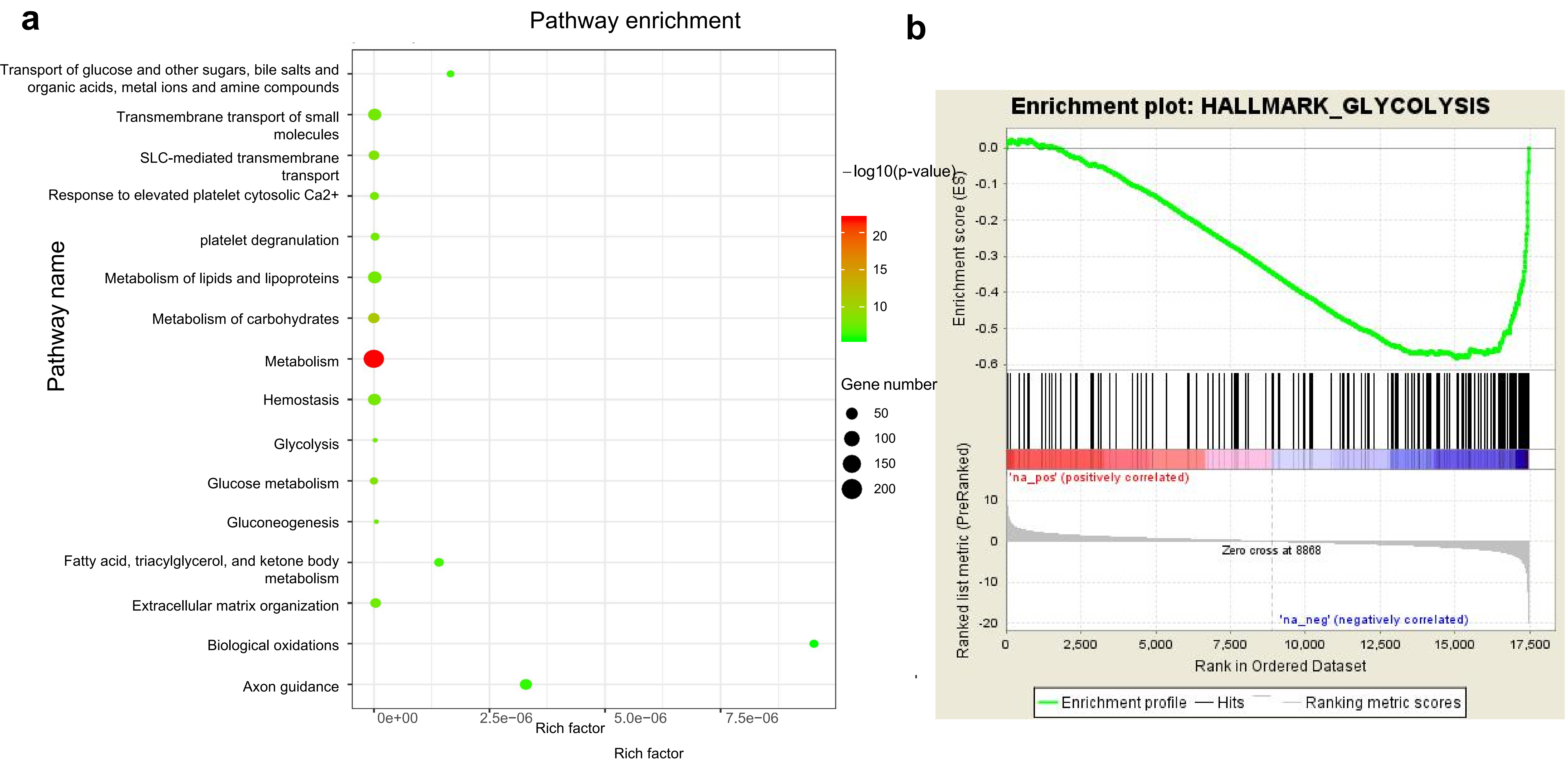

Supplementary Figure 3. MTR4 regulates the expression of metabolic genes. (a) Enrichment Pathway analysis of the RNA-Seq data from HCC cells before and after MTR4 knockdown. (b) Significant GSEA Enrichment Score curves (green curve) indicated glycolysis pathway. Genes in the red area are correlated positively with MTR4 expression, and genes in the blue area are correlated negatively with MTR4 expression. The vertical black lines indicate the position of genes listed.

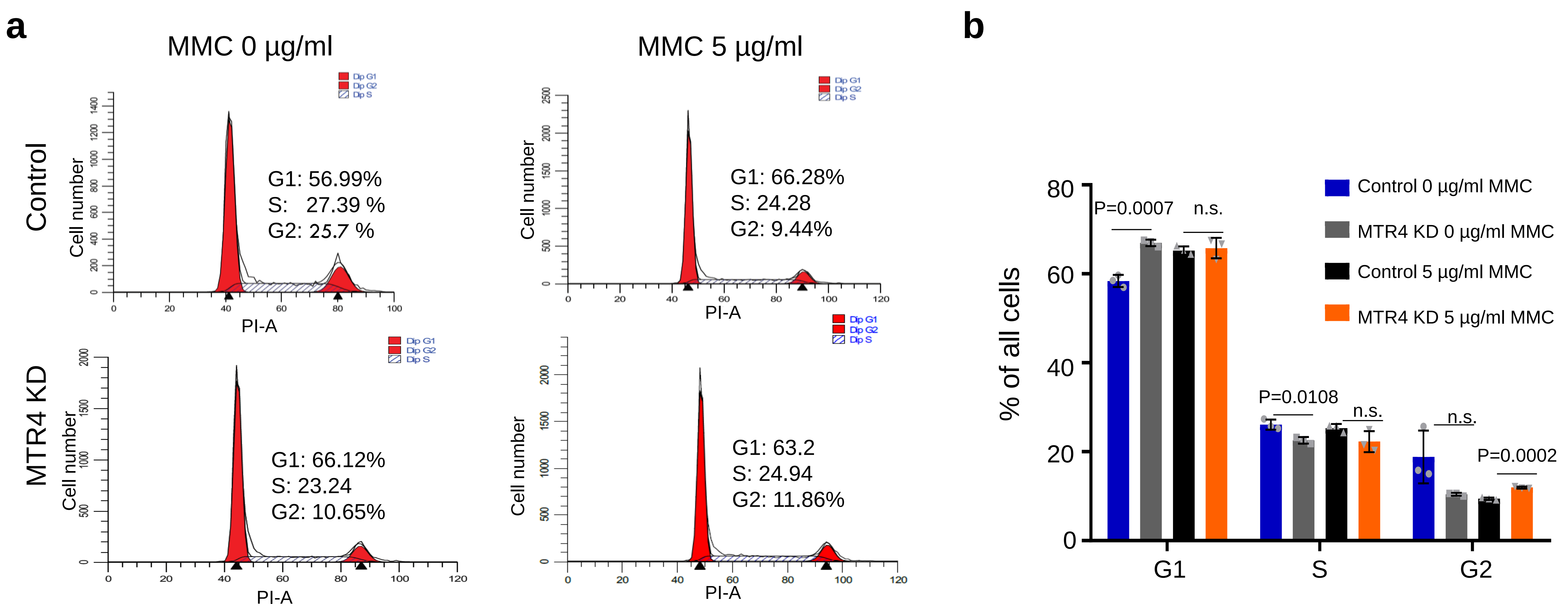

Supplementary Figure 4. Cell cycle distribution of control cells and MTR4 KD cells after treated with mitomycin C(MMC). Cell cycle analysis of cells after incubation with MMC (5µg /ml) for 12 hours. (a) Representative data on cell cycle distribution of MTR4 KO and control cells with or without MMC treatment, Cell debris and dead cells were gated out (Supplementary Figure 7). (b) Statistical analysis of cell cycle distribution of MTR4 KO and control cells with or without MMC treatment. Data are presented as mean values ± s.d. n=3 biologically independent experiments. Two-tailed, unpaired t-test. p values are indicated. n.s., non-significant

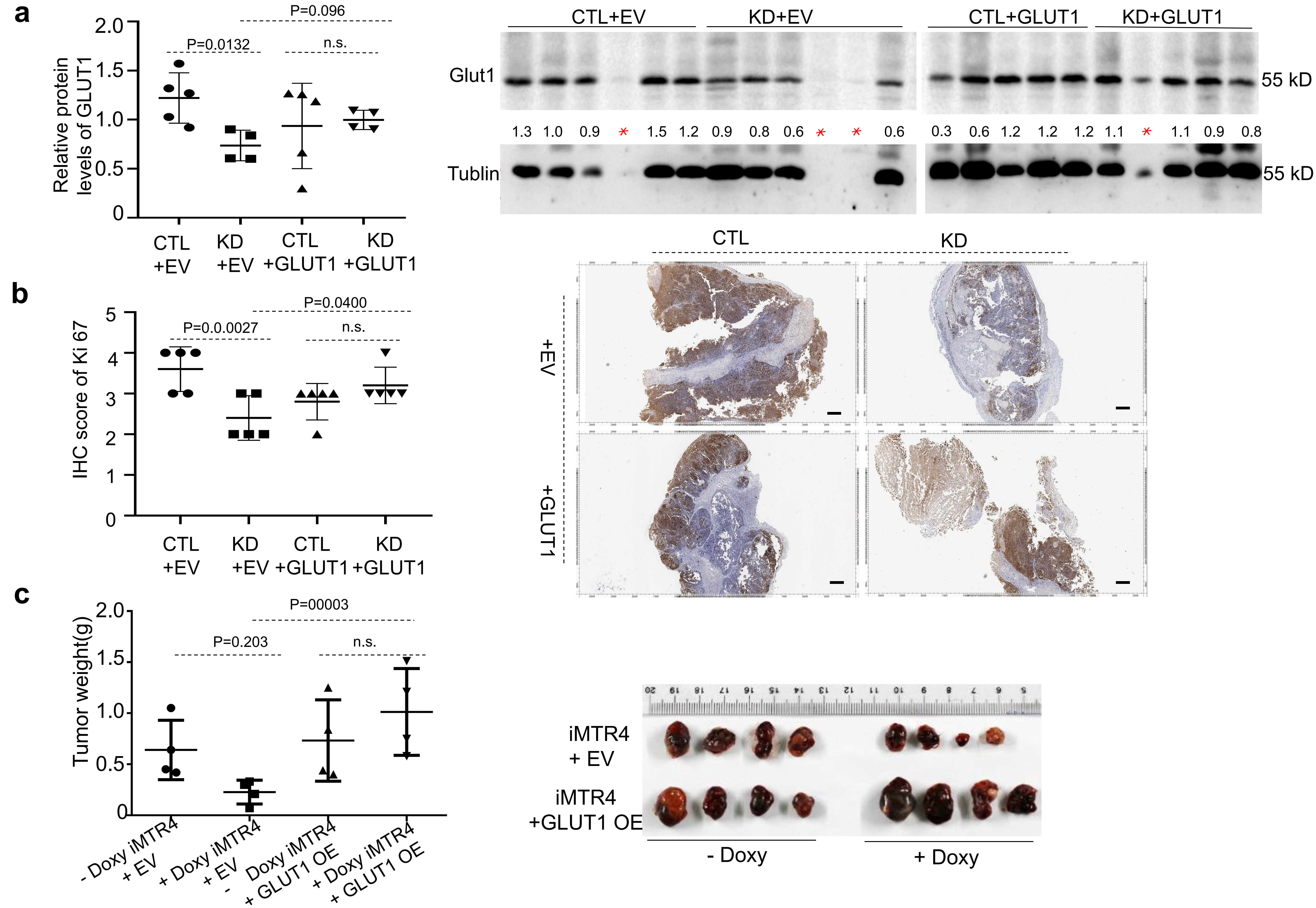

Supplementary Figure 5. Ectopic expression of GLUT1 rescued the defective growth in MTR4 KD HCC cells. (a) The protein levels of GLUT1 were reduced in the tumors formed by MTR4 KD HCCs and normalized in tumors formed by MTR4 KD HCCs ectopically expressing MTR4. The tumor samples without protein input were indicated with asterisks and excluded from the analysis. Two-way ANOVA with a Tukey's multiple comparison test. The relative expression of GLUT1 protein was normalized by the expression of tubulin protein in various tumor samples. EV, empty vector. CTL, control. KD, MTR4 knockdown. CTL+GLUT1, HCC with ectopic expression of GLUT1. KD+GLUT1, HCC with MTR4 KD and ectopic expression of GLUT1. n=5 independent tumor samples in the CTL+EV/CTL+GLUT1group, n=4 independent tumor samples in the KD+EC/KD+GLUT1 group. Data are presented as mean values ± s.d. p values are indicated. n.s., non-significant. (b) The quantification of $Ki67^{+}$ cells in various tumor samples by IHC analysis. Representative images of $Ki67^{+}$ cells in tumor sections were shown. The score of the $Ki67^{+}$ cells in various tumor samples were compared with *p* values indicated. The positively stained samples were scored as follows: 1, ≤25% of all cells; 2, 25%-≤50% of all cells; 3, 50%-≤75% of all cells; 4, ≥75% of all cells. Scale bar= 200μm， n= 5 independent tumors for each group. Data are presented as mean values ± s.d. Two-way ANOVA with a Tukey's multiple comparison test. p values are indicated on graphs. (c) Tumors formed by iMTR4 HCC in NSG mice were measured after the daily i.p. injection of doxy (20 mg/kg body weight) or PBS for 9 days. At the end of the treatment, the weight of all tumors in each group was compared. n= 4 independent tumor samples for each group. Data are presented as mean values ± s.d. Two-way ANOVA with a Tukey's multiple comparison test . p values are indicated.

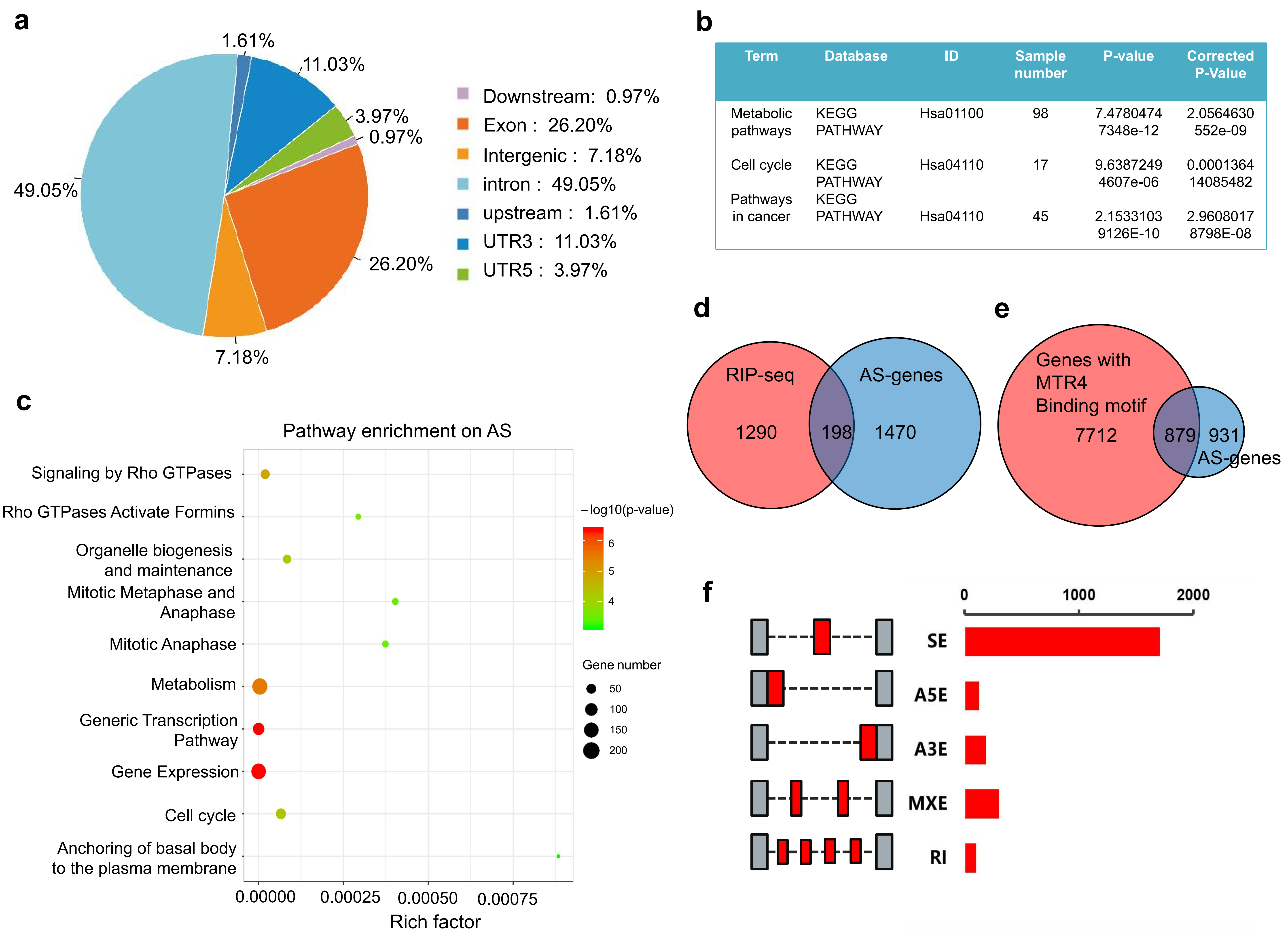

Supplementary Figure 6. MTR4 primarily binds to the intronic region of pre-mRNAs enriched in metabolic and cell cycle pathways with significant overlap with genes undergoing abnormal AS in HCC samples. (a) pre-mRNAs bound by MTR4 were identified by RIP-seq analysis. Distribution of sequencing signals bound by MTR4 within the exon, intron, intergenic, UTR3, UTR5, downstream, and upstream sequences is indicated. (b) The MTR4-bound transcripts are enriched in metabolic, cell cycle and cancer pathways. The pathway enrichment analysis was conducted by a hypergeometric statistic test. The Benjamini and Hochberg FDR correction was used to correct the probability values. (c) Genes undergoing differential AS in HCC cells after MTR4 depletion are enriched in metabolic pathways. (d, e) The overlap of the MTR4 target genes identified by RIP-seq or predicted by the presence of MTR4 binding motifs with the genes undergoing abnormal AS events after MTR4 KD. (f) Number of different AS events in HCC cells affected by MTR4 depletion.

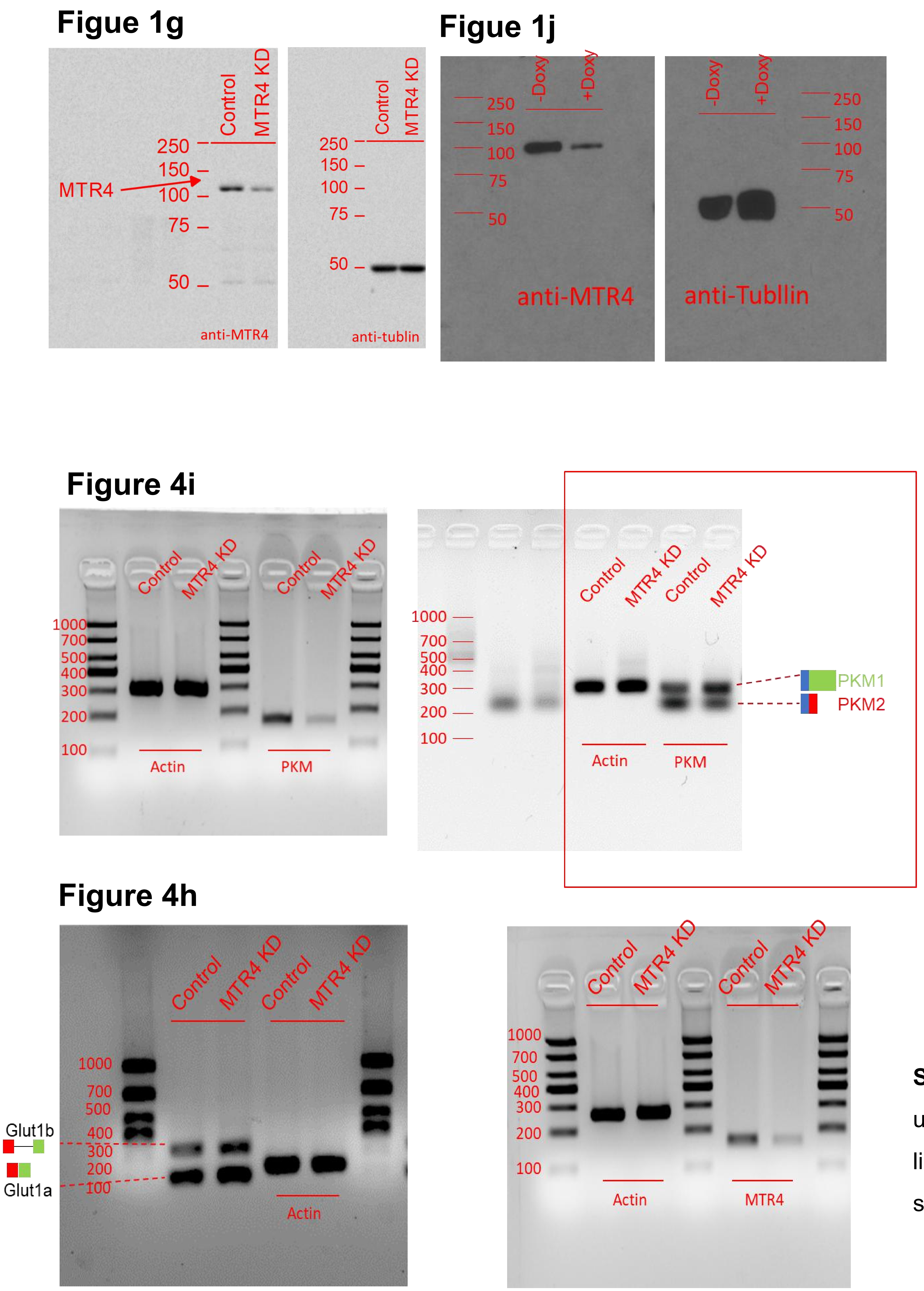

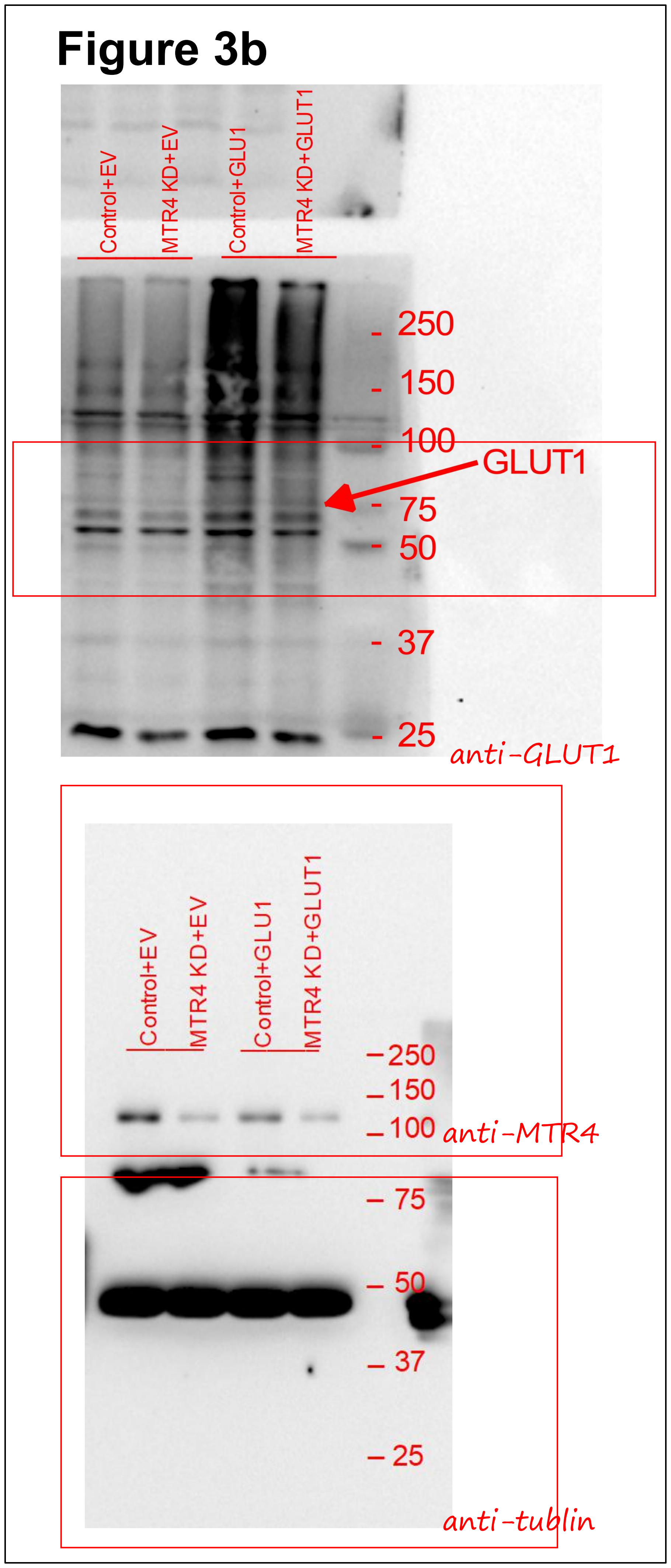

Supplementary Figure 7. Uncropped and unprocessed Western Blots or gels. Red line boxes indicate the cropped areas shown in the corresponding figures.

Figure 5a

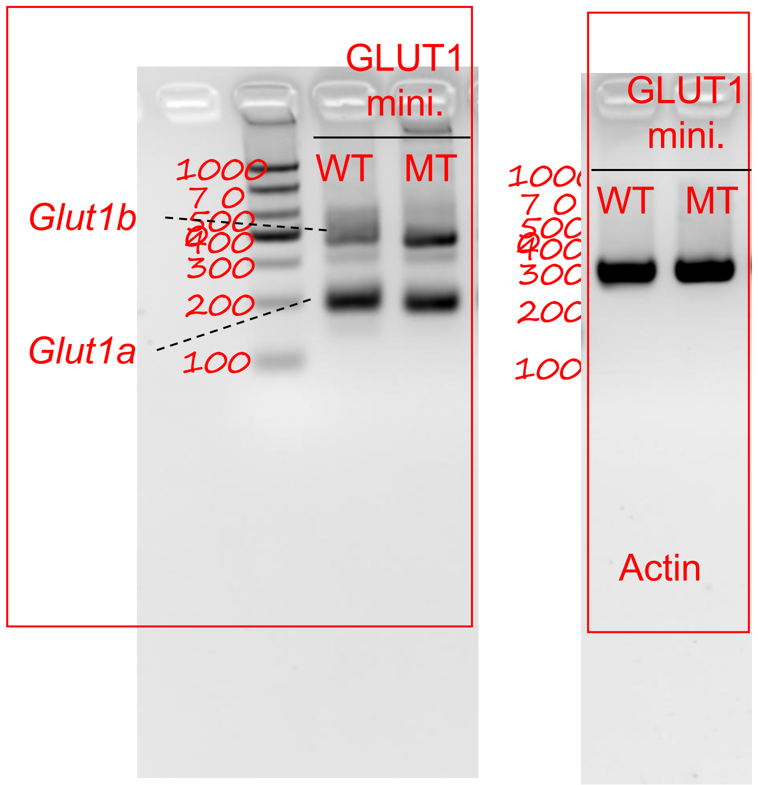

Figure 6d

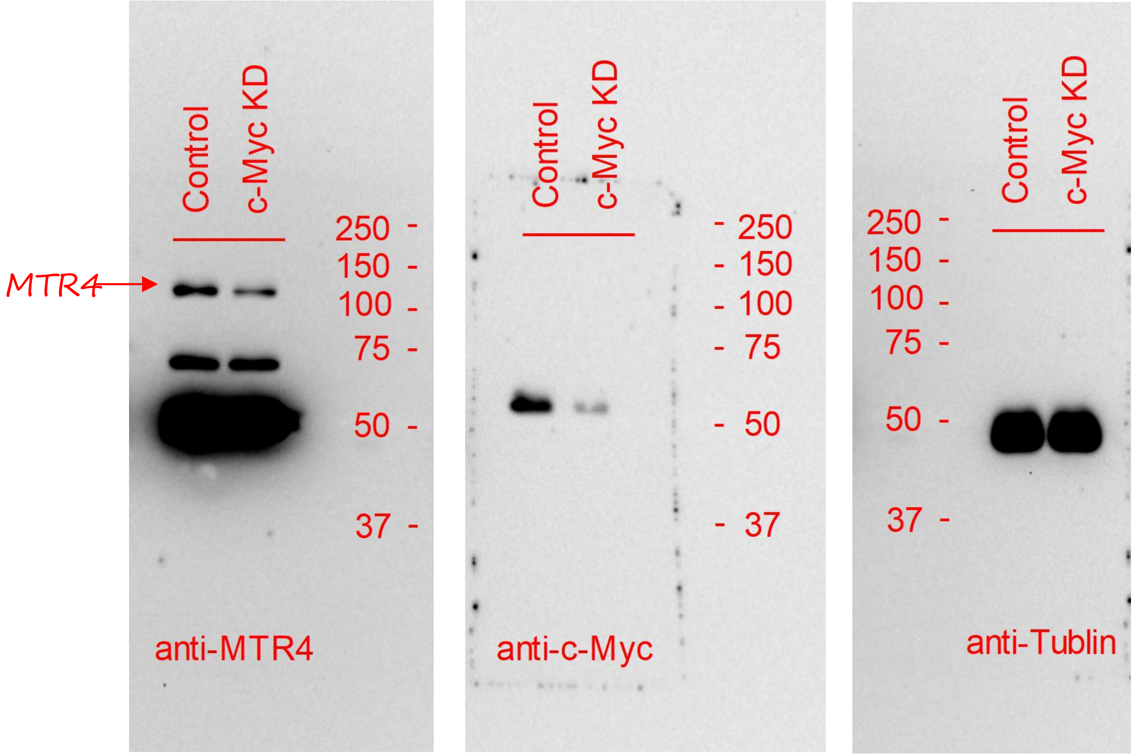

Figure 5c

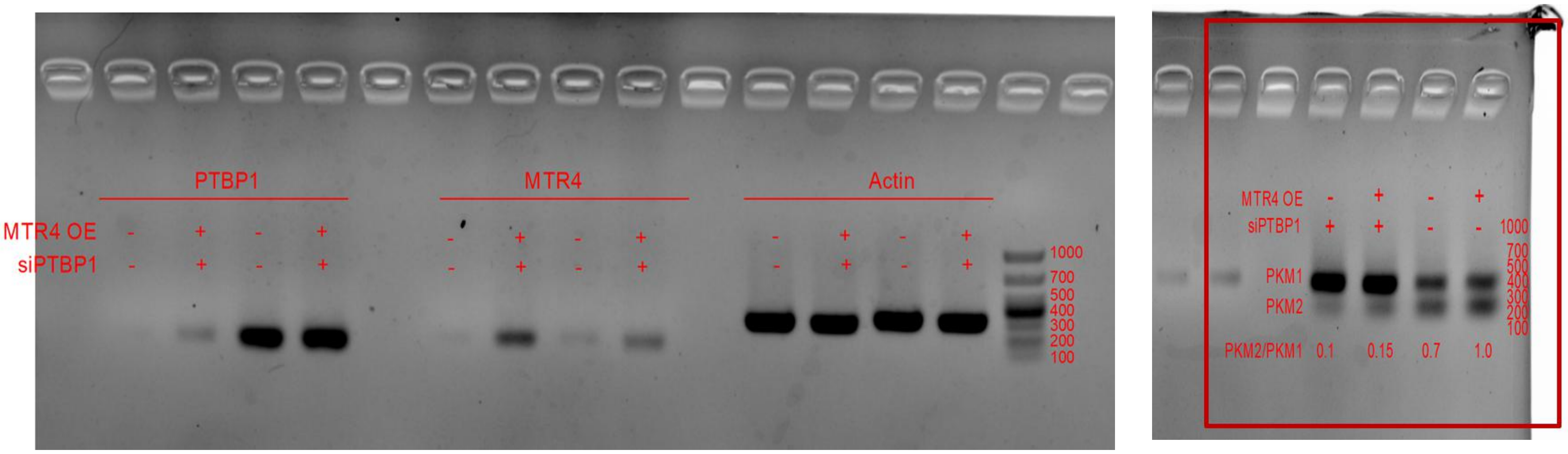

Figure 5d

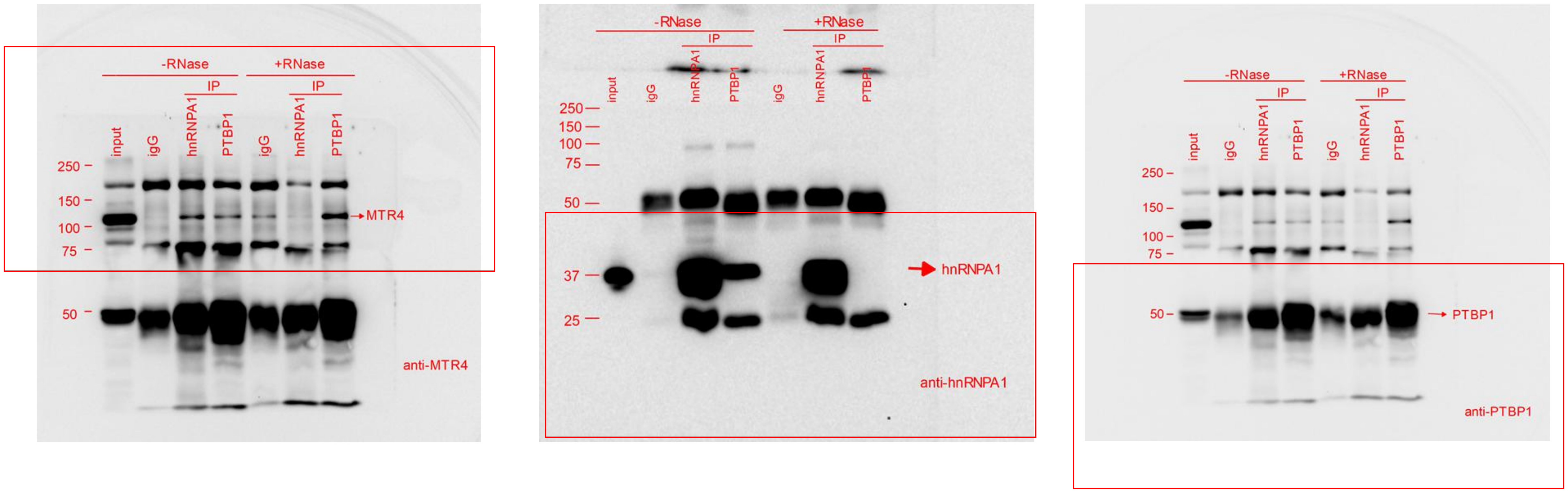

Supplementary Figure 7 continued

Figure 6e

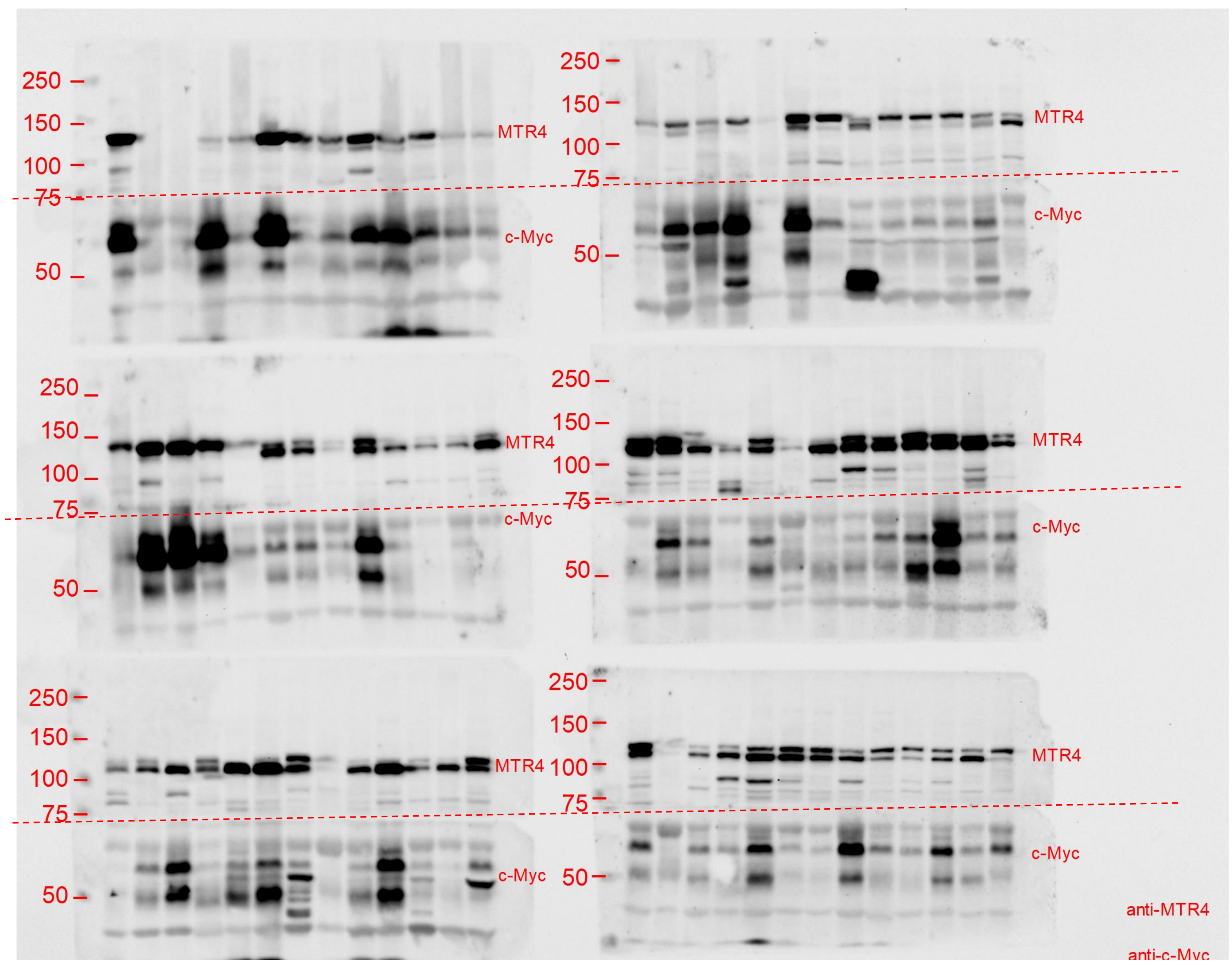

Figure S2a

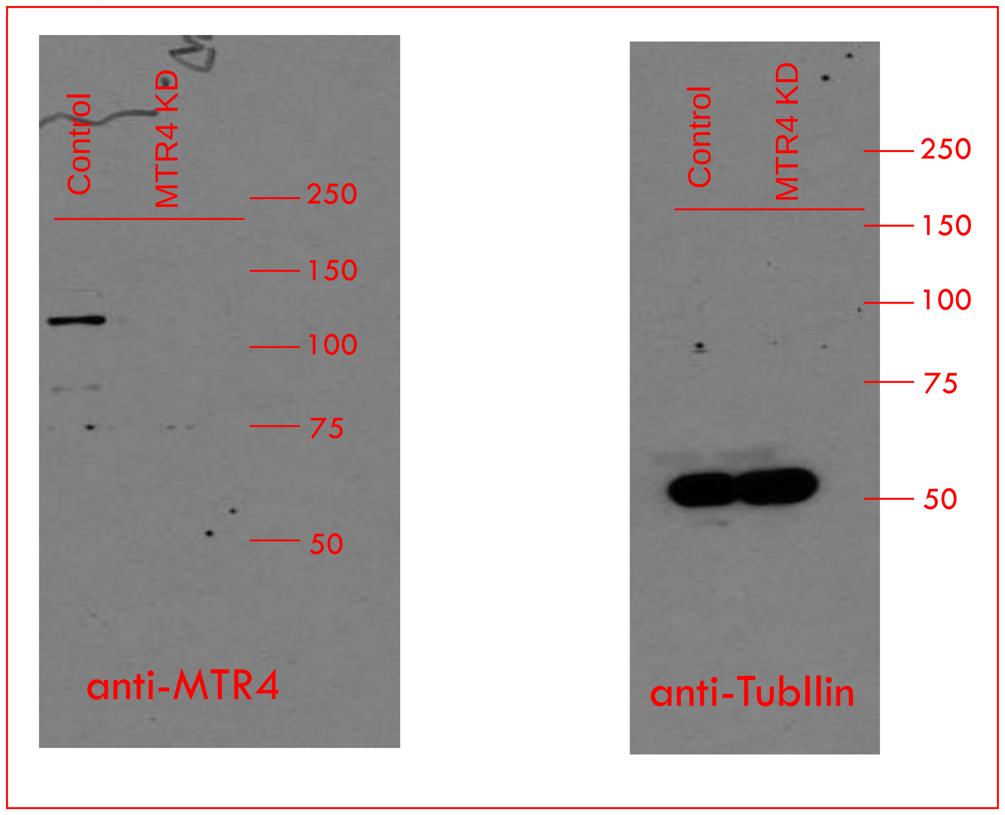

Figure S2d

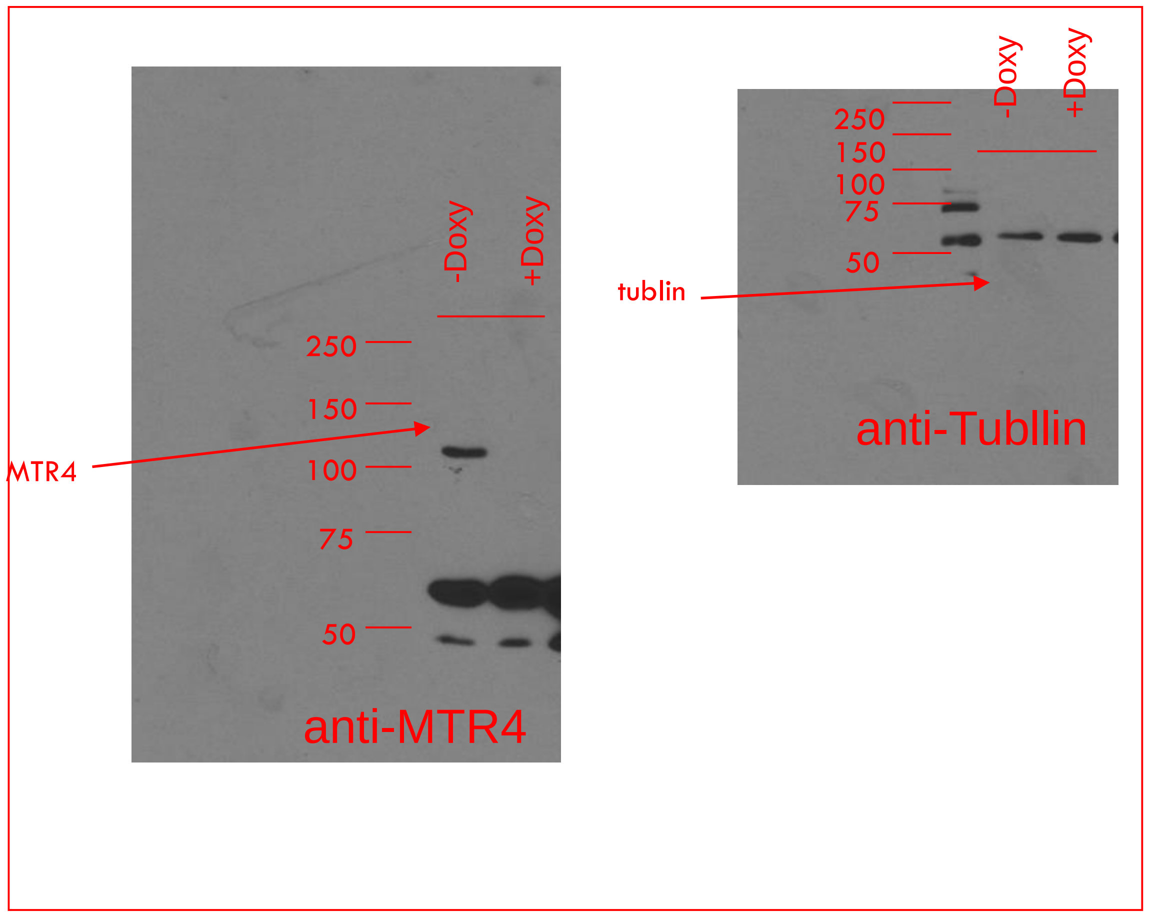

Figure S5a

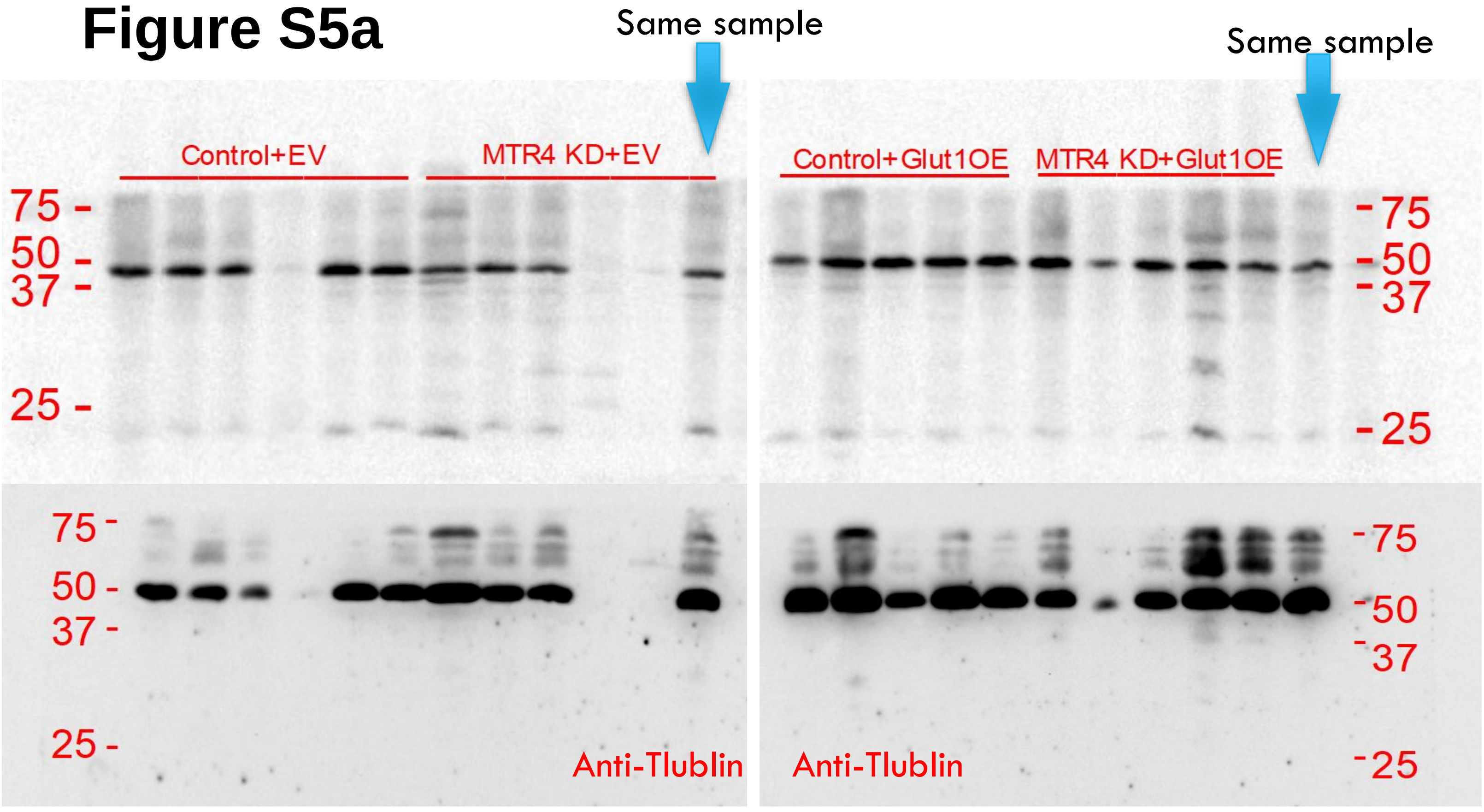

Figure S4a

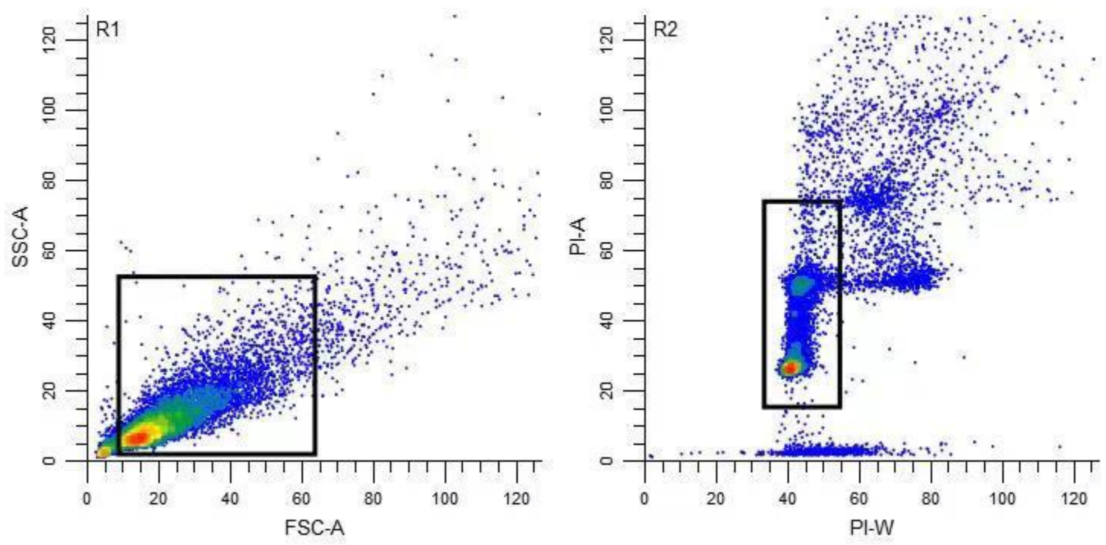

Supplementary Figure 7 continued

Figure S5b

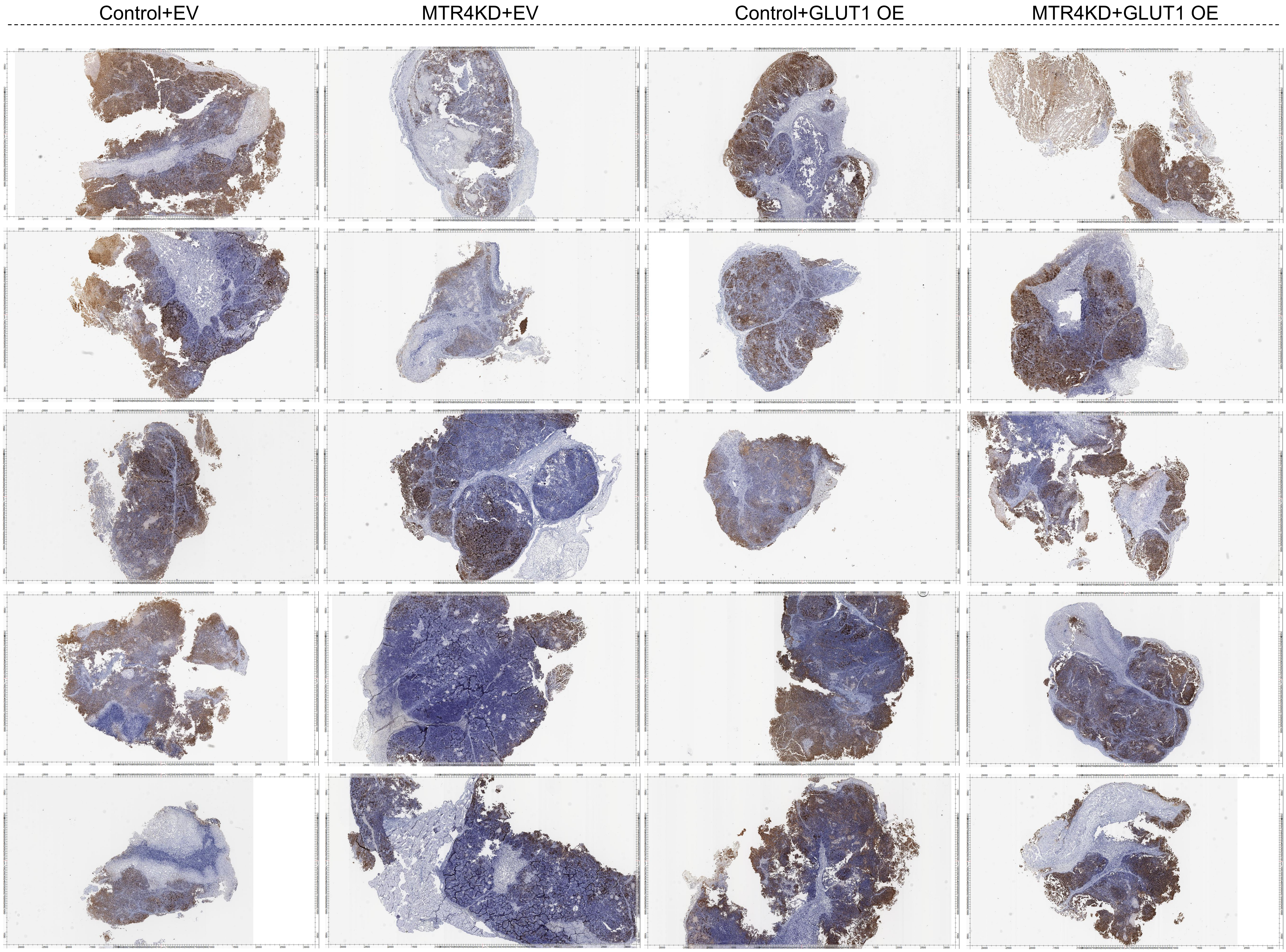

Supplementary Figure 7 continued

Supplementary Table 1. Primer sequence for qPCR

MTR4-F AACCCATTTTTGGAAAGAAGCCC

MTR4-R CAGGAAGTGCAACCTCATGTGT

GLUT1-F CTTGGCTCCCTGCAGTTTG

GLUT1-R GGACCCATGTCTGGTTGTAG

HK1-F GTCCATTCCTGATGGCTCTG

HK1-R TCTCATGATTCACTTGCACCC

HK2-F TTGACATGGGCTCACTGAAC

HK2-R CATCTTCACCAGGATAAGCCTC

GPI-F TGCCCTGTCTACTAACACAACC

GPI-R GCAGCTGCTCGAAGTTGTCA

PFKL-F TGGACCTGGAGAAGCTGC

PFKLR ACATAAATGCCCATGCGCG

LDOA-F GCGGTGTTGTGGGCATCAAG

LDOA-R CAGACAGCCCATCCAACCCT

GAPDH-F GAAGGTGAAGGTCGGAGTC

GAPDH-R CATGTAAACCATGTAGTTGAGGTC

PGAM1-F GAACCTGGAGAACCGCTTCA

PGAM1-R GATGTCAAACTCATAGCCAGCA

ENO1-F CTTTACGTTCACCTCGGTGTC

ENO1-R TCTCCCTGGCATGGATCTTG

ENO2-F CAGGACTTTGTCAGGGACTATC

ENO2-R CAGGTCATCACCCACAATCT

PKM2 F (ex10) ATTATTTGA GGA ACT CCGCCGCCT

PKM2-R (exon11) ATTCCGGGTCACAGCAATGATGG

PKM1-F (exon9) CGAGCCTCAAGTCACTCCAC

PKM1-R (exon11) GTGAGCAGACCTGCCAGAC

LDHA-F CTTCTAAAGGAAGAACAGACCC

LDHA-R CAAGAGCAAGTTCATCTGCC

Actin-F AGCGAGCATCCCCCAAAGTT

Actin-R GGGCACGAAGGCTCATCATT

Supplementary Table 2. Primer sequence for RIP and CHIP analysis.

RIP peak 2416 intron 9 F CAAAGTGACACCCTGTCTCAAA

RIP peak 2416 intron 9 R GCCAGGCCCAGTCTACATTA

RIP peak 2415 intron 10 F GCAACACTCCCATTTCCTGT

RIP peak 2415 intron 10 R CCCAGCAACTGGGCTATAAA

RIP3 peak 27 stop codon F AAACATGGCTGTGTTTGCAG

RIP3 peak 27 stop codon R CAGTCCAGCATTCCTCCTTC

RIP3 peak 29 start point F AATTTGTCTCGACCCAGGAC

RIP3 peak 29 start point R CATGTGCTCCAGGAATGTGT

CHIP Promoter F1 TGAGATGGTGCTCAGGATGA

CHIP Promoter R1 TGACAGTAGCCCTGTTGCAG

CHIP Promoter F3 GGGAGATTTGCTCTCACTGC

CHIP Promoter R3 TTCCCCTTGTCCTTTTCCTT

CHIP Promoter motif F8 CGTGTAAAGTGCCTAACAGTGG

CHIP Promoter motif R8 GTGGCTGTATGCACCAGTCT

Supplementary Table 3. Primer sequence for AS analysis of minigenes.

Mini Glut1 exon7 F CGTCAACACGGCCTTCACTGT

Mini WPRE R CATAGCGTAAAAGGAGCAACA

Mini PKM AS exon8 F CTATCCTCTGGAGGCTGTGC

Mini PKM AS exon9 R TGCCAGACTCCGTCAGAACT

Mini PKM AS exon8-exon10 R GGCAATCAGGTGCTGCAT

Glut1 mini gene-RIP (intron)F TGACACTGTCTCTGCCCACA

PKM mini gene-RIP (exon11) F ACCTGTACCGTGGCATCTTC
