# Supplementary material for: MTR4 drives liver tumorigenesis by promoting cancer metabolic switch through alternative splicing
Source: Nat Commun. 2020 Feb 5;11:708. doi: 10.1038/s41467-020-14437-3 (PMC7002374; doi:10.1038/s41467-020-14437-3)
Supplement: Supplementary file 2 — Reporting Summary [file 41467_2020_14437_MOESM2_ESM.pdf]

nature research

Corresponding author(s): NCOMMS-18-19445D DBPR

Last updated by author(s): Dec 20, 2019

Reporting Summary

Nature Research wishes to improve the reproducibility of the work that we publish. This form provides structure for consistency and transparency in reporting. For further information on Nature Research policies, see Authors & Referees and the Editorial Policy Checklist .

Statistics

For all statistical analyses, confirm that the following items are present in the figure legend, table legend, main text, or Methods section.

n/a	Confirmed	
☐	☒	The exact sample size (*n*) for each experimental group/condition, given as a discrete number and unit of measurement
☐	☒	A statement on whether measurements were taken from distinct samples or whether the same sample was measured repeatedly
☐	☒	The statistical test(s) used AND whether they are one- or two-sided<br>*Only common tests should be described solely by name; describe more complex techniques in the Methods section.*
☐	☒	A description of all covariates tested
☐	☒	A description of any assumptions or corrections, such as tests of normality and adjustment for multiple comparisons
☐	☒	A full description of the statistical parameters including central tendency (e.g. means) or other basic estimates (e.g. regression coefficient) AND variation (e.g. standard deviation) or associated estimates of uncertainty (e.g. confidence intervals)
☒	☐	For null hypothesis testing, the test statistic (e.g. *F*, *t*, *r*) with confidence intervals, effect sizes, degrees of freedom and *P* value noted<br>*Give P values as exact values whenever suitable.*
☒	☐	For Bayesian analysis, information on the choice of priors and Markov chain Monte Carlo settings
☒	☐	For hierarchical and complex designs, identification of the appropriate level for tests and full reporting of outcomes
☐	☒	Estimates of effect sizes (e.g. Cohen's *d*, Pearson's *r*), indicating how they were calculated

Our web collection on statistics for biologists contains articles on many of the points above.

Software and code

Policy information about availability of computer code

Data collection	N/A- no custom algorithm or software was used
Data analysis	N/A- no custom algorithm or software was used

For manuscripts utilizing custom algorithms or software that are central to the research but not yet described in published literature, software must be made available to editors/reviewers. We strongly encourage code deposition in a community repository (e.g. GitHub). See the Nature Research guidelines for submitting code & software for further information.

Data

Policy information about availability of data

All manuscripts must include a data availability statement. This statement should provide the following information, where applicable:

- Accession codes, unique identifiers, or web links for publicly available datasets
- A list of figures that have associated raw data
- A description of any restrictions on data availability

The sequencing datasets were deposited in the GEO and are now available.

Field-specific reporting

Please select the one below that is the best fit for your research. If you are not sure, read the appropriate sections before making your selection.

☒ Life sciences ☐ Behavioural & social sciences ☐ Ecological, evolutionary & environmental sciences

For a reference copy of the document with all sections, see nature.com/documents/nr-reporting-summary-flat.pdf

Life sciences study design

All studies must disclose on these points even when the disclosure is negative.

Sample size	The sample size was chosen based on our experience and published studies on the same type of experiments. Sample size for each experiment is indicated in the figure legend. No statistical method was used to predetermine sample size.
Data exclusions	No data was excluded.
Replication	All experiments were repeated with reproducible data. The number of experimental repeats is reported in figure legends.
Randomization	Samples were randomly allocated into different groups.
Blinding	The investigators were blinded to group allocation during the collection and analysis of patient's sample and their clinical data.

Reporting for specific materials, systems and methods

We require information from authors about some types of materials, experimental systems and methods used in many studies. Here, indicate whether each material, system or method listed is relevant to your study. If you are not sure if a list item applies to your research, read the appropriate section before selecting a response.

Materials & experimental systems

n/a	Involved in the study
☐	☒ Antibodies
☐	☒ Eukaryotic cell lines
☒	☐ Palaeontology
☐	☒ Animals and other organisms
☐	☒ Human research participants
☐	☒ Clinical data

Methods

n/a	Involved in the study
☒	☐ ChIP-seq
☐	☒ Flow cytometry
☒	☐ MRI-based neuroimaging

Antibodies

Antibodies used	rabbit polyclonal anti-MYC (13987, Cell signaling)<br>rabbit polyclonal anti-MTR4 (ab70551, Abcam)<br>anti-Glut1 (ab150299, Abcam)<br>mouse monoclonal anti–α-tubulin (T5168, Sigma-Aldrich)<br>anti–rabbit IgG, HRP-linked antibody (7074S, Cell Signaling Technology)<br>anti–mouse IgG, HRP-linked antibody (7076S, Cell Signaling Technology)<br>mouse monoclonal anti-hnRNPA1 03-204, Millipore)<br>mouse monoclonal anti-PTBP1(MABE-986, Millipore)
Validation	All antibodies used in the study are commercially available and validation statement is provided on manufacturer's website.

Eukaryotic cell lines

Policy information about cell lines

Cell line source(s)	293FT cells were obtained from Thermo Fisher Scientific. HepG2 and PLC/PRF/5 cells were obtained from ATCC.
Authentication	The cell lines were purchased from the authorized source and authenticated. HepG2 cells was authenticated by Cell line Authentication Service from Biowing in China and PLC/PRF/5 cells was authenticated by Aiji.
Mycoplasma contamination	All cell lines were tested negative for mycoplasma contamination.
Commonly misidentified lines (See ICLAC register)	No cell lines used in this study is listed on the ICLAC list of commonly misidentified cell lines.

Animals and other organisms

Policy information about studies involving animals; ARRIVE guidelines recommended for reporting animal research

Laboratory animals	NSG mice

Wild animals	This study did not involve wild animals.
Field-collected samples	This study did not involve sample collected from the field.
Ethics oversight	All animal work was approved by Institutional Animal Care and Use Committee (IACUC) of Southern Medical Univeristy and University of California, San Diego.

Note that full information on the approval of the study protocol must also be provided in the manuscript.

Human research participants

Policy information about studies involving human research participants

Population characteristics	All patients satisfied the following inclusion criteria: they had not received any other treatments before this surgery; HBV-related HCC, without hepatitis C virus (HCV) infection, was diagnosed pathologically and serologically; the surgical margins were confirmed to contain no residual carcinoma tissue; complete medical records were available. Clinicopathological information on age, gender, tumor size, tumor number, tumor capsule, differentiation, AFP, Cirrhosis, MVI, PVTT, TNM and BLCL stage characteristics are provided in Table 1.
Recruitment	HCC tissues and corresponding noncancerous tissues were obtained from consecutive treatment-naive HBV-related HCC patients who underwent curative resection for HCC in the Department of Hepatobiliary Surgery, Nanfang Hospital Affiliated to Southern Medical University, Guangzhou, China, between November 2010 and May 2015.
Ethics oversight	This study was approved by IRB of Nanfang Hospital at Southern Medical University and performed according to the Declaration of Helsinki (6th revision, 2008)

Note that full information on the approval of the study protocol must also be provided in the manuscript.

Clinical data

Policy information about clinical studies

All manuscripts should comply with the ICMJEguidelines for publication of clinical research and a completedCONSORT checklist must be included with all submissions.

Clinical trial registration	*Provide the trial registration number from ClinicalTrials.gov or an equivalent agency.*
Study protocol	*Note where the full trial protocol can be accessed OR if not available, explain why.*
Data collection	*Describe the settings and locales of data collection, noting the time periods of recruitment and data collection.*
Outcomes	*Describe how you pre-defined primary and secondary outcome measures and how you assessed these measures.*

Flow Cytometry

Plots

Confirm that:

- [x] The axis labels state the marker and fluorochrome used (e.g. CD4-FITC).
- [x] The axis scales are clearly visible. Include numbers along axes only for bottom left plot of group (a 'group' is an analysis of identical markers).
- [x] All plots are contour plots with outliers or pseudocolor plots.
- [x] A numerical value for number of cells or percentage (with statistics) is provided.

Methodology

Sample preparation	Cells were seeded onto 6-well plates at a density of 1×106 cells/well and treated with MMC (5μg /ml) for 12 hours. Cells were fixed with cold 70% ethanol for 24 hr at 4 °C and incubated with 0.5 mg/ml of propidium iodide (PI) along with 0.1 mg/ml of RNase A (MultiSicences, China).
Instrument	FACScan, BD Biosciences
Software	ModFIt LTV4.1.7
Cell population abundance	After gating, only the live cells remained for analysis. The percentage of cells within each cell cycle phase was indicated.
Gating strategy	Cell debris and dead cells were gated out for analysis.

- [x] Tick this box to confirm that a figure exemplifying the gating strategy is provided in the Supplementary Information.
